# Target Capture of Ancient Shell DNA Enables Phylogenetic Reconstruction of Deep‐Sea Molluscs

**DOI:** 10.1111/1755-0998.70199

**Published:** 2026-08-30

**Authors:** Yi‐Xuan Li, Yanjie Zhang, Qi Dai, Chong Chen, Crispin T. S. Little, Moriaki Yasuhara, Yusuke Yokoyama, Jack Chi‐Ho Ip, Jian‐Wen Qiu

**Affiliations:** ^1^ Department of Biology Hong Kong Baptist University Hong Kong SAR China; ^2^ School of Life and Health Sciences, Hainan Province Key Laboratory of One Health, Collaborative Innovation Center of One Health Hainan University Haikou China; ^3^ X‐STAR, Japan Agency for Marine‐Earth Science and Technology (JAMSTEC) Yokosuka Kanagawa Japan; ^4^ School of Earth, Environment and Sustainability University of Leeds Leeds UK; ^5^ Life Sciences Department Natural History Museum London UK; ^6^ School of Energy and Environment City University of Hong Kong Hong Kong SAR China; ^7^ Atmosphere and Ocean Research Institute The University of Tokyo Chiba Japan; ^8^ Division of Science Lingnan University Hong Kong SAR China

**Keywords:** aDNA, Bivalvia, deep sea, Pliocardiinae, ultraconserved elements, Vesicomyidae

## Abstract

Target capture is widely used to enrich endogenous DNA from calcium phosphate skeletal material in vertebrates, but its performance on calcium carbonate hard parts widely produced by invertebrates remains poorly understood. Here, we compared DNA recovery from four fresh and 12 ancient (eight radiocarbon‐dated to 1671–1135 years old before present) deep‐sea vesicomyid clam shells, including species *Archivesica marissinica*, *A. nanshaensis* and *A. okutanii*, using whole‐genome sequencing (WGS) or target capture of ultraconserved elements (UCEs). WGS achieved 16.65% on‐target read recovery of UCEs from fresh soft tissue, but < 1% from shell specimens. By contrast, UCE capture in the same specimen increased on‐target reads by up to 155‐fold, reaching 29.84% in fresh shells and up to 72‐fold, reaching 19.89% in ancient shells. Target capture of UCEs recovered 142–1001 loci per sample compared to 0–230 with WGS alone. Ancient shells of *A. marissinica* and *A. okutanii*, based on reads mapped with bwa‐mem2 and bbmap, exhibited characteristic post‐mortem DNA damage signals, with average 5′‐end C‐to‐T misincorporation rates of 3.46% and 15.97%, respectively, exceeding the levels observed in fresh *A. marissinica* shells (maximum 1.24%). UCE‐based phylogenetic reconstructions incorporating shell ancient DNA recovered two major clades within Pliocardiinae, consistent with published phylogenomic trees. Together, these findings demonstrate that target‐capture enrichment enables effective recovery of highly degraded DNA from ancient mollusc shells and supports robust phylogenetic inference at the intrageneric scale, expanding the utility of shells—one of the most abundant invertebrate remains—for evolutionary, biogeographic and conservation studies.

## 1 Introduction

While palaeogenomics in vertebrate animals has become a routine practice for generating genome‐scale data from ancient bones and teeth (Leonardi et al. 2017; Keighley et al. 2021; Chen et al. 2025), vast marine biological archives from invertebrate animals remain comparatively untapped (Armbrecht et al. 2019; Green and Speller 2017; Wang et al. 2021). Among these, molluscan shells are especially abundant across archaeological and fossil records, serving as vital proxies for palaeoenvironmental dating, temperature and salinity (Kádár and Costa 2006; Bau et al. 2010; Watanabe et al. 2012; Markulin et al. 2019). During shell formation, endogenous DNA can be incorporated into both intracrystalline and intercrystalline organic matrices, though the intracrystalline fraction is less susceptible to diagenetic decay and offers a more reliable molecular window into the past (Marin and Luquet 2004). However, extracting this genomic information poses severe technical challenges. Unlike dense vertebrate bones that preserve DNA effectively (Campos et al. 2012), molluscan shells comprise 95%–99% calcium carbonate and only 1%–5% organic matrix (Marin and Luquet 2004), resulting in exceedingly low endogenous DNA yields. Consequently, early shell DNA studies were largely restricted to short, PCR‐amplified markers for species identification (Geist et al. 2008; Ferreira et al. 2020), without fully realizing the immense potential of shell DNA for broad ecological and evolutionary inferences. More recently, high‐throughput whole‐genome sequencing (WGS) has been applied to historical and ancient shells to bypass the limitations of PCR (Der Sarkissian et al. 2017, 2020; Sullivan et al. 2021; Martin‐Roy et al. 2024). Yet, given the low endogenous DNA content, WGS on shell substrates remains prohibitively expensive and highly inefficient for recovering informative nuclear loci.

Target capture sequencing based on the Illumina sequencing platform has gained popularity for processing highly degraded samples, such as historical museum samples (~100–500 years before present, BP) and ancient remains (> 1000 years BP, Buenaventura 2021; Salter et al. 2022). However, its application to marine organisms is exceedingly rare (Kirch et al. 2021). To date, only a single study has employed target capture on old molluscan shells, retrieving mitochondrial genomes from abalone shells up to 60 years old (Walton et al. 2023). While this highlights the feasibility of the target capture approach, its efficacy remains untested on ancient shells. Subfossil biominerals (~1000–10,000 years BP) have undergone vastly different preservation conditions compared to museum collections, yet they often represent the primary biological archives or the sole accessible material for resolving the evolutionary history of rare lineages.

Deep‐sea vesicomyid clams (Venerida: Vesicomyidae) are one of the most dominant animal groups in deep‐sea chemosynthetic ecosystems, exemplified by hydrothermal vents and hydrocarbon seeps (Johnson et al. 2017). However, the evolutionary relationships of taxa within the subfamily Pliocardiinae remain contentious due to sparse sampling of live material, while some species are known exclusively from empty shells (Decker et al. 2012; Johnson et al. 2017; Gao et al. 2025). Genome‐scale nuclear data are key in resolving the phylogeny of such lineages but remain unavailable for many taxa. A previous work underscored the limitation of WGS for shell aDNA, as testing three pairs of ~1500‐year‐old *Archivesica nanshaensis* shells (Li et al. 2023) only recovered 12 mitochondrial contigs and 2913 nuclear contigs. As such, there is a need to develop a more efficient, targeted approach.

Hybridization capture of ultraconserved elements (UCEs) is an appealing solution. UCEs offer applicability across different evolutionary scales (McCormack et al. 2016; Branstetter et al. 2017; Stiller et al. 2021), and probe sets have recently been developed for bivalves (Goulding et al. 2023; González‐Delgado et al. 2024; Li et al. 2025; Batistão et al. 2025; Chen et al. 2026), showing high cross‐taxon transferability for modern tissue samples. Here, we integrate UCE target capture with aDNA, testing the ‘Bivalve UCE 2k v.1’ probe set (captured ~2000 UCE loci, Li et al. 2025) on radiocarbon‐dated ancient shells of three vesicomyid clam species: *Archivesica marissinica*, *A. okutanii* and *A. nanshaensis*. Leveraging available nuclear and mitochondrial references for vesicomyids, we aimed to compare the efficiency of UCE capture versus WGS on *A. marissinica* shells, characterize post‐mortem damage patterns across different deep‐sea environments (seep vs. vent), and evaluate the phylogenetic utility of shell‐derived loci.

## 2 Materials and Methods

### 2.1 Sampling and Radiocarbon Dating

**FIGURE 1:**
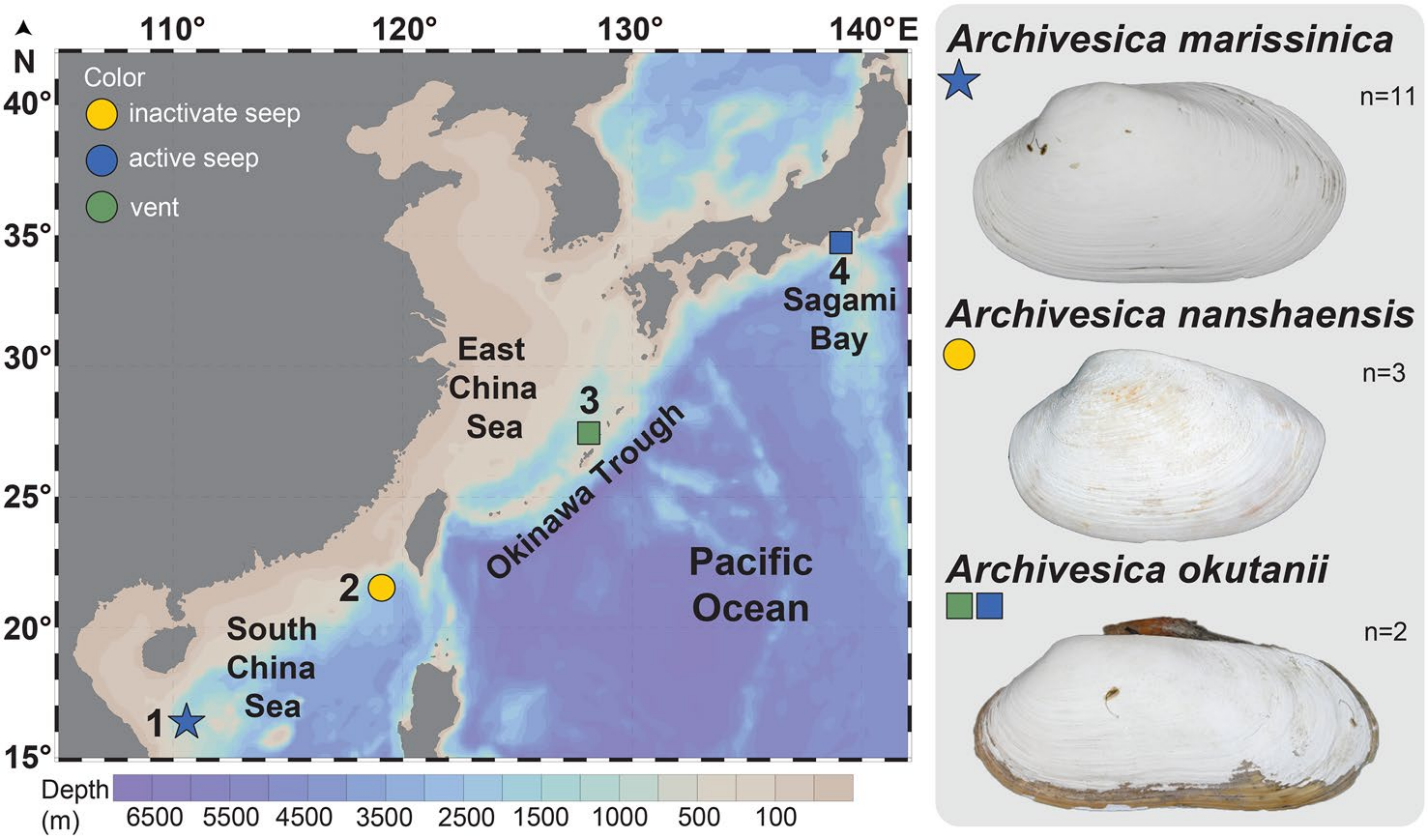
Sampling information of deep‐sea vesicomyid clam shells. 1, Haima seep, South China Sea; 2, Inactive seep, Northern South China Sea; 3, Sakai Field, Okinawa Trough; 4, Off Hatsushima, Sagami Bay; n, number of paired shell specimens. The map was generated using Ocean Data View (ODV) v.5.0 (https://odv.awi.de). Details of shells in Table 1 and Figures S1–S4.

*Archivesica marissinica*, *A. nanshaensis* and *A. okutanii* shells were collected by remotely operated vehicles (ROVs) between 2015 and 2020 from two active hydrocarbon seeps (Haima seep in the South China Sea, the Off Hatsushima seep in Sagami Bay, Japan), an unnamed inactive seep in the northern South China Sea, and the Sakai hydrothermal vent field in the Okinawa Trough (Figures 1, S1–S4, and Table 1). The studied shell samples do not originate from protected species. Four articulated pairs of fresh shells from *A. marissinica* and nine ancient shells from three species were stored in a dark environment at −80°C (specimens Amar1–4), −20°C (specimens Amar5–9, transferred from −80°C), or a dry room (~25°C for specimens Amar10–11, Anan1–3, and Aoku1–2). Due to limitations in the available material, we only compared fresh versus ancient shell DNA for *A. marissinica* samples. Four valves from four specimens of *Archivesica marissinica* and two valves from two *Archivesica okutanii* shells were radiocarbon‐dated (^14^C) by Accelerator Mass Spectrometry (AMS). Approximately 500 μg of shell material from one randomly selected valve for each specimen was processed at the Atmosphere and Ocean Research Institute, the University of Tokyo, or at Beta Analytic (USA). The radiocarbon dates were calibrated to calendar ages by OxCal 4.4.4 (Ramsey 2021) using the Marine20 dataset (Heaton et al. 2020) and the global standard reservoir age (ΔR = 0). We applied conservative ΔR, given the scarcity of empirical regional ΔR for the deep‐sea area near our sampling sites.

**TABLE 1:** Sample collection information. F, fresh shell. A, ancient shell. Age (Cal years BP), calendar year before present. #1–4, Marked in Figure 1: #1 Haima methane seep, South China Sea, #2 Inactive seep, Northern South China Sea, #3 Sakai Vent Field, Okinawa Trough, #4 Off Hatsushima seep, Sagami Bay [1], whole genome sequencing. [2] target‐capture sequencing. *, Data from Li et al. (2023). Details of radiocarbon dating for shells in Table S1.

Species	Individual ID	Type	Collection date	Age (Cal years BP)	Region	Coordinates	Depth (m)	Sequencing
*Archivesica marissinica*	Amar1	F	2018/05/13	NA	#1	N16.69° E110.39°	1361	[1]
	Amar2	F	2020/09/02	NA	#1	N16.69° E110.39°	1360	[1], [2]
	Amar3	F	2020/09/02	NA	#1	N16.69° E110.39°	1360	[1], [2]
	Amar4	F	2020/09/02	NA	#1	N16.69° E110.39°	1360	[1], [2]
	Amar5	A	2021/05/18	NA	#1	N16.26° E111.83°	1385	[2]
	Amar6	A	2021/05/18	1584–1305	#1	N16.26° E111.83°	1385	[1], [2]
	Amar7	A	2021/05/18	1675–1370	#1	N16.26° E111.83°	1385	[2]
	Amar8	A	2019/05/07	NA	#1	N16.26° E111.83°	1380	[1]
	Amar9	A	2019/05/07	NA	#1	N16.26° E111.83°	1380	[1]
	Amar10	A	2019/05/07	1405–1160	#1	N16.26° E111.83°	1380	[1]
	Amar11	A	2019/05/07	1812–1530	#1	N16.26° E111.83°	1380	[1]
Archivesica nanshaensis*	Anan1	A	2018/05/09	1616–1340	#2	N21.35° E120.98°	3035	[1]
	Anan2	A	2018/05/09	1618–1341	#2	N21.35° E120.98°	3035	[1]
	Anan3	A	2018/05/09	NA	#2	N21.35° E120.98°	3035	[1]
*Archivesica okutanii*	Aoku1	A	2015/07/29	1326–1074	#3	N35.58° E141.31°	1605	[1]
	Aoku2	A	2016/06/30	1270–1001	#4	N27.45° E128.1°	907	[1]

### 2.2 Shell DNA Extraction and Library Preparation

We conducted experiments for fresh and ancient shells in separate clean rooms, using personal protective equipment to avoid contamination. Total genomic DNA was extracted from sieved shell powder (0.6–0.8 g) with a blank control, which was dissolved in 30 mL 0.5 M EDTA buffer (pH = 8.0) and gently rotated for 24 h at room temperature. After centrifugation (5000 × g, 10 min), pellets were resuspended in 2 mL 2× CTAB buffer (100 mM Tris–HCl in pH 8.0, 1.4 M NaCl, 20 mM EDTA in pH 8.0, 2% CTAB, 2% PVP40), split into two Eppendorf tubes, and incubated overnight at 60°C with 40 μL proteinase K (20 mg/mL). Supernatants were then subjected to phenol‐chloroform extraction in a UV‐sterilized, HEPA‐filtered, dust‐free laminar flow hood to minimize contamination, as in Li et al. (2023). Shell DNA was dissolved in TE buffer and concentrated to 25 μL using a Concentrator Plus vacuum centrifuge (Eppendorf, Hamburg, Germany) at room temperature for library preparation.

Due to the availability of both modern and ancient shells, as well as genomic resources (Ip et al. 2021), our study mainly focused on *A. marissinica*. The ancient shells with different lengths and at least one complete valve were randomly selected for the enrichment experiment. To assess the capture efficiency of UCEs on degraded shell DNA, target‐capture enrichment was applied to three fresh‐shell (Amar2–4) and three ancient *A. marissinica* shell samples (Amar5–7) with the Bivalve UCE 2 k v.1 probe set designed to target ~2000 UCEs based on high‐quality bivalve genomes and transcriptomes, including *A. marissinica* (Li et al. 2025). Shell DNA libraries were prepared without size selection using Invitrogen Collibri PS DNA Library Prep Kit for Illumina Systems with unique dual indexes (Thermo Fisher Scientific, MA, USA), followed by PCR amplification (16 cycles) and purification using magnetic beads and reagents within the same kit. Library concentrations were measured with a Qubit 4 fluorometer (Thermo Fisher Scientific, MA, USA) using the Qubit dsDNA HS Assay (Thermo Fisher Scientific, MA, USA). Three capture pools spanning the six libraries were each concentrated to 7 μL and hybridized at 60°C with the Bivalve UCE 2 k v.1 probes, following the manufacturer's optimized protocol for highly degraded DNA (myBaits Manual v5.03). Post‐capture samples were purified using clean‐up reagents from the library preparation kit used above, then quantified using a Qubit 4 fluorometer, quantitative PCR (qPCR), and a 2100 Bioanalyzer (Agilent, CA, USA).

Non‐captured shell DNA libraries (nine *A. marissinica* and two *A. okutanii*) were prepared using the NEB Next Ultra DNA Library Prep Kit (New England Biolabs, MA, USA) at Novogene (Tianjin, China). Captured and non‐captured libraries were sequenced on an Illumina Novaseq 6000 at Novogene (Tianjin, China) to generate 150 bp paired‐end reads. Three fresh‐shell (Amar2–4) and one ancient shell (Amar6) samples were subjected to both WGS and UCE target capture sequencing to allow comparison.

### 2.3 Raw Data Processing

Raw reads were demultiplexed by unique dual indexes and trimmed with Trimmomatic v.0.38 (Bolger et al. 2014) under the following settings: “Illuminaclip:TruSeq3‐PE; fa:2:30:10; Leading:3; Trailing:3; Slidingwindow: 4:15; Minlen:20”. Read quality was assessed with FastQC v.0.11.9 (Brown et al. 2017). Putative viral, bacterial and vector contaminants were removed using bbduk.sh (BBMap v.0.38; Bushnell 2014).

### 2.4 Detection of Target Reads

Two mapping approaches were used to recover reads matching *A. marissinica* nuclear (GenBank GCA_014843695.1, Ip et al. 2021) and mitochondrial (GenBank OM397546) genomes: (1) bwa‐mem2 v.2.2.1 (Li 2013) and samtools v.1.19 (Danecek et al. 2021), followed by duplicate removal using Picard v.2.27.5 (BroadInstitue 2019); (2) BBMap's bbmap.sh under default settings. To avoid redundancy, we merged unfiltered mapped reads per library (bam file with the same LB label) and removed duplicates using Picard. For *A. okutanii* shells, we applied an assembled *A. okutanii* transcript as the reference (GenBank accession number GIAT00000000.1). To compare data composition across specimen types (tissue, fresh shell, ancient shell) and sequencing strategies (whole genome sequencing vs. target capture), combined and non‐redundant nuclear bam files were re‐formatted to fastq files using samtools and then examined using COMMET v.1.0 (Maillet et al. 2014) with K = 32.

Captured efficiency was evaluated by comparing effective UCE reads of target‐capture and non‐captured data. The 5% of the *A. marissinica* tissue genome survey data (SRR11675938) were randomly sampled for comparison. The *A. marissinica* UCE reference was extracted from the genome (GCA_014843695.1) using PHYLUCE v.1.7.2 (Faircloth 2016) against Bivalve UCE 2 k v.1 probes (sets v1 and v2). UCE‐mapped reads were obtained with bwa‐mem2 and samtools, filtered using Picard, summarized using samtools and visualized in R with ggplot2 (Wilkinson 2011). Statistical comparisons of mapped reads, on‐target read rates and depth between ancient and fresh shell samples or WGS and captured data were performed using the two‐sided Mann–Whitney U test (Wilcoxon rank‐sum test) at Statistics Kingdom (https://www.statskingdom.com/170median_mann_whitney.html).

### 2.5 DNA Damage Analysis

BBMap‐mapped and bwa‐mem2‐mapped reads were further filtered using samtools to retain MAPQ30 reads for DNA damage assessment. Fresh‐shell data were analysed directly with PMDtools v.1.1 (Skoglund et al. 2014). For ancient shells, no filtration or PMD score thresholds of 1 or 3 were applied to remove potential modern contaminants before estimating damage (Table S2), following PMDtools recommendations.

### 2.6 Data Assembly and UCE Loci Recovery

Both *de novo* and reference‐guided assembly strategies were employed as in Li et al. (2023). For *de novo* assembly, clean reads for each dataset were assembled using SPAdes v.3.15 (Nurk et al. 2013) under default settings for paired‐end data. For reference‐guided assembly, BBMap‐mapped reads were assembled with SPAdes using ‐‐only‐assembler and ‐‐cov‐cutoff off, respectively. Contigs were queried by BLASTn (BLAST v.2.13; e‐value 1e‐10) against *A. marissinica* references (genome: GCA_014843695.1; mitochondrial genome: OM397546) and *A. okutanii* (mitochondrial genome: AP014555). Contigs were discarded with < 80% identity to reference sequences. Assemblies of each sample were merged and deduplicated using BBMap's dedupe.sh (k‐mer = 17, min_match = 20, min_identity = 98). Public metagenomic and genome‐skimming datasets (Table S3) were *de novo* assembled using SPAdes under the same settings. Transcriptomes (Table S3) were assembled with Trinity v.2.8.5 (Grabherr et al. 2011) under default settings. UCE loci were extracted from each assembly using PHYLUCE v.1.7.2 with Bivalve UCE 2 k v.1 probes (sets v1 and v2) (Li et al. 2025).

### 2.7 Phylogenetic Analysis

UCE loci and mitochondrial (mt) contigs were used to assess the phylogenetic signal. Three UCE‐ and one mt‐concatenated matrices were built with different outgroups: *Matrix‐A* (species outside Vesicomyidae), *Matrix‐B* (*Ruditapes philippinarum* as outgroup), *Matrix‐C* (expanded Pliocardiinae transcriptome data) and *Matrix‐D* (Pliocardiinae mt genome data only). Each UCE partition was aligned with MAFFT v.7.475 (Katoh and Standley 2013) using “phyluce_align_seqcap_align” and edge‐trimming. Given the limited UCEs recovery from WGS, *Matrix‐A* (1,246 loci; 377,654 bp) and *Matrix‐B* (1,036 loci; 528,248 bp) were constructed without taxon‐occupancy filtering. *Matrix‐C* (20% species occupancy; 488 loci, 127,617 bp) was generated using the transcriptome‐derived probe set (v2). Mitochondrial dataset *Matrix‐D* (11,441 bp) concatenated 13 protein‐coding gene alignments (PCGs) trimmed with trimAl v.1.4.1 (Capella‐Gutiérrez et al. 2009) using the “automated1” setting. Modelfinder v.1.5.4 (Kalyaanamoorthy et al. 2017) selected a substitution model per partition, and Maximum‐Likelihood (ML) trees were inferred using IQ‐TREE2 v.2.2.7 (Minh et al. 2020) with 5000 ultrabootstrap replicates. Trees were visualized with tvBOT (Xie et al. 2023) with annotations (Table S4–S6).

## 3 Results

### 3.1 Radiocarbon Dating, Sequencing Outputs and Mapping Results

Radiocarbon analysis confirmed that the old shell collections of *A. marissinica* and *A. okutanii* represent ancient materials, as well as *A. nanshensis* (Li et al. 2023). Calibrated ages of four *Archivesica marissinica* valves were dated back to 1812–1160 years BP (Tables 1 and S1), comparable to *A. nanshaensis* (1618–1340 years BP), but older than *A. okutanii* collections (1326–1001 years BP). The δ^13^C_PDB_ (AMS) and IRMS δ^13^C values of shell samples were from −1.4‰ ± 0.9‰ to 1.1‰ ± 0.5‰ (Table S1). The near‐zero δ^13^C values indicated that the source of carbon for shell formation in *A. marissinica* and *A. okutanii* was distinctly different from methane‐derived dissolved inorganic carbon; therefore age determination of these shells should not be affected by the dead‐carbon effect that might confound the age determination using the ^14^C method (Adkins et al. 2002; Ambrose et al. 2015).

WGS generated 54.17 to 209.79 million raw reads per library, while UCE target capture generated 33.97 to 167.12 million raw reads per library (Table 2). After trimming, WGS retained an average of 91.92% clean reads (Table 2), although Ama5 had low quality (30.64%). Target capture retained 56.07%–96.03% clean reads. Contaminant filtering removed 1.09%–16.70% (0.58–27.2 million) of reads from WGS, and 5.21%–9.28% (1.67–8.92 million) of reads were filtered from UCE‐captured datasets (Table 2). Data composition was more similar within sequencing strategies (WGS vs. target‐captured sequencing) than across sample types (tissue, fresh shell, ancient shell) (Figure S5).

**TABLE 2:** Statistics of sequencing and mapping of shell DNA after deduplication. F, fresh shell. A, ancient shell. Raw reads, raw sequencing reads. Clean reads, reads retained after quality filtering. Contaminants, reads mapped to bacteria, virus and vector sequences. Nuclear and mitochondrial reads, non‐redundant reads mapped to *Archivesica marissinica* nuclear and mitochondrial genome, respectively. Duplicates%, proportion of duplicates between bwa‐mem2 and bbmap mapped nuclear reads. Duplicates%*, proportion of duplicates between bwa‐mem2 mapped and bbmap mapped mitochondrial reads. #, target capture sequencing data.

Specimen	Type	Raw reads	Clean reads	Contaminants	Nuclear reads	Duplicates%	Mitochondrial reads	Duplicates (%)*
*Archivesica marissinica*								
Amar1	F	75,818,260	74,816,628	122,7928	2,803,778	12.30	1947	48.22
Amar2	F	209,794,668	204,364,280	3,259,089	29,632,137	33.98	7137	50.20
Amar3	F	173,065,974	168,665,900	2,132,954	16,858,797	27.88	16,900	61.26
Amar4	F	169,289,240	164,978,600	2,662,053	13,972,367	32.29	39,281	61.82
Amar2#	F	33,970,122	32,002,158	1,666,398	739,338	98.51	59	38.54
Amar3#	F	149,187,346	98,788,696	5,566,453	2,389,621	98.76	272	64.55
Amar4#	F	167,121,996	93,710,938	5,652,206	1,848,157	98.75	956	68.10
Amar5#	A	103,341,834	96,218,514	8,923,809	1,875,685	98.19	174	66.67
Amar6	A	197,940,588	193,393,824	4,580,260	4,813,438	20.20	361	57.67
Amar6#	A	71,882,686	69,221,550	4,418,048	1,714,010	69.11	165	68.63
Amar7#	A	60,056,798	56,386,852	5,229,960	403,296	97.61	61	47.41
Amar8	A	167,818,998	163,419,168	27,285,860	5,311,401	30.93	364	58.03
Amar9	A	183,209,744	56,136,202	5,973,629	439,975	92.51	20	NA
Amar10	A	72,790,952	72,000,044	2,944,480	3,440,455	24.41	1070	53.17
Amar11	A	72,366,752	71,657,582	1,664,799	2,280,291	16.77	80	29.20
*Archivesica okutanii*								
Aoku1	A	72,783,206	71,388,466	2,271,818	4,976,478	18.60	141	42.91
Aoku2	A	54,171,924	53,275,728	578,498	15,589,284	22.30	3549	41.85

**FIGURE 2:**
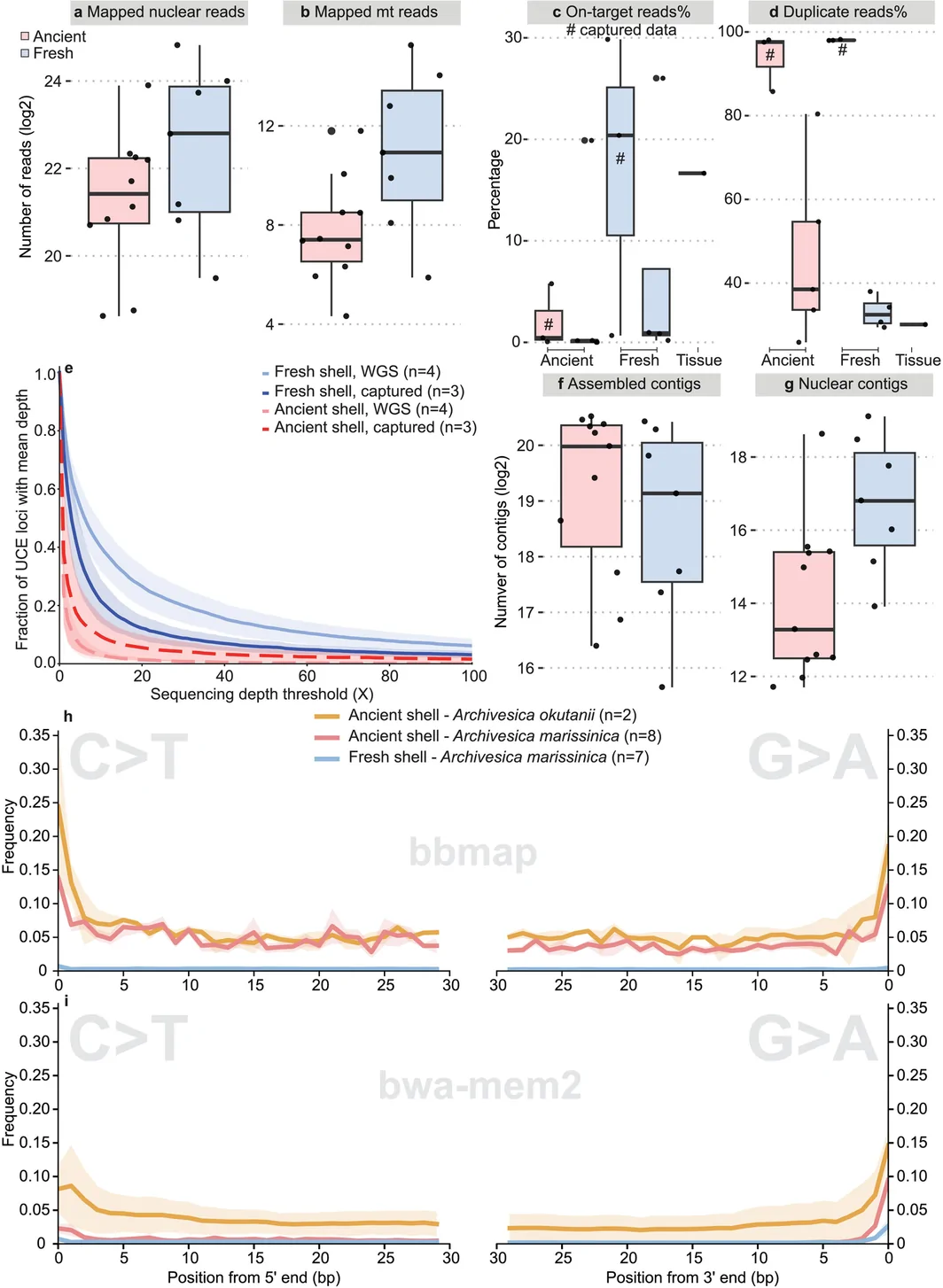
Summary of sequencing data for deep‐sea shells against the genome and mitochondrial genome of *Archivesica marissinica*. (a, b) mapped nuclear and mitochondrial reads from all fresh (*A. marsiinica*) and ancient shells (*A. marissinica* and *A. okutanii*); (c) on‐target reads rate of *A. marissinica* shells mapped to 1627 UCEs; (d) duplicate reads rate of *A. marissinica* shells mapped to 1627 UCEs; (e) mean depth of reads mapped to representative 1627 UCEs from *A. marissinica*, WGS, whole‐genome sequencing data; captured, UCE target‐capture sequenced data; (f, g) assembled contigs and filtered nuclear contigs from all fresh (*A. marissinica*) and ancient (three species) shells; (h, i) DNA damage of fresh (*A. marissinica*) and ancient (*A. marissinica* and *A. okutanii*) shells based on captured and non‐captured bbmap‐mapped data (h) and bwa‐mem2‐mapped data (i). #, UCE target captured data. Tissue, WGS data sequenced using fresh soft tissue, randomly selected 5% from SRR11675938.

In *A. marissinica*, fresh shells yielded more mapped nuclear (0.74–29.63 million vs. 0.40–5.31 million) and mitochondrial reads (59–39,281 vs. 20–1070) than ancient shells (Table 2, Figure 2a,b). Fresh shells exhibited significantly higher mapped read percentages than ancient shells when applying WGS (*p* < 0.05, Table S7). Across species, ancient *A. okutanii* shells yielded higher mapped outputs (nuclear: 4.00–15.58 million vs. 0.40–5.31 million; mitochondrial: 141–3549 vs. 20–1070) than ancient *A. marissinica* shells (Table 2, Figure 2b–f). Captured datasets showed higher duplication for nuclear reads (69.11%–98.76%) than WGS data (12.3%–33.98%) (Table 2); mitochondrial duplicate rates were similar between UCE‐capture (38.54%–68.63%) and WGS (29.20%–61.82%). For *A. marissinica*, WGS shell data showed significantly higher percentage of mapped reads compared to target‐captured data (*p* < 0.01, Table S7).

We quantified UCE capture efficiency by comparing on‐target read recovery from *A. marissinica* soft tissue and shell (targeting 1627 UCE loci across 1,547,522 bp; Table 3). Fresh shell samples had significantly higher on‐target UCE reads rates than ancient shell samples (*p* < 0.05, Table S7). WGS recovered a significantly lower proportion of UCE reads (*p* < 0.01, Table S7): 0.19%–0.97% in fresh shells and 0.009%–0.18% in ancient shells (Figure 2c and Table 3). In contrast, UCE capture achieved 20.38%–29.84% on‐target rates in fresh shells and 0.45%–19.88% in ancient shells with lower input (Figure 2c and Table 3). Target capture significantly enriched on‐target UCE reads compared to WGS in ancient shell samples (median: 5.78% vs. 0.06%, *p* < 0.05, Table S7). High enrichment efficiency was observed in fresh shell and ancient shell specimens after enrichment (Tables 3, 4), 26–155 fold over WGS data of the same specimen (Figure 2d), with increased duplicated fractions (85.80%–98.20% vs. 25.90%–80.40% in WGS) and improved coverage at 10× depth (1.16%–23.64% vs. 0.12%–7.21% in WGS) in ancient shells (Figures 2e, S6 and S7).

**TABLE 3:** Statistics of mapping WGS and UCE target‐capture sequences against *Archivesica marissinica* UCEs (1627 UCEs, 1,547,522 bp). S, soft tissue. F, fresh shell. A, ancient shell. Clean reads, reads available for mapping. Mapped reads, mapped reads without deduplication. NA, not applicable for enrichment comparison of the same specimen.

Specimen	Data type	Type	Clean reads	Mapped reads	On‐target reads (%)	Fold enrichment
Ama_SCS001	WGS	S	30,090,300	5,011,527	16.65	NA
Amar1	WGS	F	74,816,628	639,405	0.85	NA
Amar2	WGS	F	204,364,280	1,976,790	0.97	26.89
Amar2	Target‐captured	F	32,002,158	8,326,823	26.02	
Amar3	WGS	F	168,665,900	324,555	0.19	155.09
Amar3	Target‐captured	F	98,788,696	29,482,044	29.84	
Amar4	WGS	F	164,978,600	1,113,024	0.67	30.22
Amar4	Target‐captured	F	93,710,938	19,106,342	20.39	
Amar6	WGS	A	193,393,824	12,067	0.006	72.177
Amar6	Target‐captured	A	69,221,550	311,744	0.45	
Amar5	Target‐captured	A	96,218,514	19,136,844	19.89	NA
Amar7	Target‐captured	A	56,386,852	3,257,431	5.78	NA
Amar8	WGS	A	163,419,168	102,429	0.06	NA
Amar9	WGS	A	56,136,202	98,351	0.17	NA
Amar10	WGS	A	72,000,044	117,219	0.16	NA
Amar11	WGS	A	71,657,582	6355	0.009	NA

**TABLE 4:** Characteristics of on‐target UCE reads from WGS and target‐sequenced shell DNA. Reference: *Archivesica marissinica* UCEs (1627 UCEs, 1,547,522 bp). S, soft tissue. F, fresh shell. A, ancient shell. Mapped reads, number of mapped reads after deduplication. Duplicates%, percentage of duplicate reads. Mean depth×, weighted mean depth, summary of all targeted UCEs (mean depth * length)/total bases. Coverage%, recovered bases versus all UCEs; 1×, 5×, 10×%: Proportion of covered bases at 1×, 5× and 10× depths.

Specimen	Data	Type	Mapped reads	Duplicates (%)	Mean depth×	Coverage%	1×%	5×%	10×%
Ama_SCS001	WGS	S	3,501,952	30.12	172.87	88.06	99.27	82.53	72.33
Amar1	WGS	F	443,091	30.70	21.54	42.77	76.05	51.13	38
Amar2	WGS	F	1,225,783	37.99	56.79	58.12	91.14	69.52	57.79
Amar2	Target‐captured	F	172,099	97.93	7.45	28.56	48.08	16.55	9.59
Amar3	WGS	F	228,881	29.48	8.79	33.22	77.03	34.33	18.81
Amar3	Target‐captured	F	588,536	98.00	26.79	68.24	94.14	57.12	35.37
Amar4	WGS	F	732,110	34.22	33.82	50.24	87.72	61.39	46.06
Amar4	Target‐captured	F	344,265	98.19	15.45	52.41	77.46	35.37	19.85
Amar6	WGS	A	7419	38.52	0.39	8.31	7.64	0.92	0.31
Amar6	Target‐captured	A	44,306	85.79	1.07	8.33	8.31	2.38	1.16
Amar5	Target‐captured	A	465,753	97.57	19.11	59.44	84.79	38.12	23.64
Amar7	Target‐captured	A	61,925	98.09	2.85	18.89	20.53	5.13	3.48
Amar8	WGS	A	67,995	33.62	2.44	20.9	39.65	8.98	4.03
Amar9	WGS	A	19,286	80.39	0.4	0.75	1.77	0.49	0.49
Amar10	WGS	A	86,875	25.89	3.7	23.67	47.59	16	7.21
Amar11	WGS	A	2881	54.66	0.12	2.27	1.16	0.12	0.12

### 3.2 Assembly Results

*De novo* and reference‐guided assemblies were generated for each shell sequencing data, then merged, and duplicates were removed. There were 65.79 thousand to 1.49 million total contigs per dataset, with only an average of 14.18% classified as target nuclear contigs (Table 5). Ancient shells produced many contigs overall but fewer target nuclear contigs (145–403,175) than fresh shells (15,388–565,073) (Figure 2f,g, Table 5).

**TABLE 5:** Assembly results of shell sequencing data. ^, *De novo* assemblies. Nuclear contigs, non‐redundant contigs mapped to *Archivesica marssinica* genome from *de novo* assemblies and ref‐based assemblies; Redundance (%), proportion of duplicated contigs and short contigs fully aligned within a longer contig. Mt genes, recovered mitochondrial genes (complete genome^#^) or gene fragments. *, data from Li et al. (2023).

Specimen	Data	Total contigs^	Total length (bp)^	Nuclear contigs	Redundance (%)	Total length (bp)	Mt genes
*Archivesica marissinica*							
Amar1	WGS	519,319	296,578,037	221,875	27.29	59,990,413	37^#^
Amar2	WGS	1,128,918	501,101,708	565,073	37.95	160,829,442	37^#^
Amar3	WGS	891,377	597,007,359	114,155	28.92	27,895,418	37^#^
Amar4	WGS	1,320,737	768,869,110	362,031	32.63	98,353,098	37^#^
Amar2	Target‐captured	65,793	18,327,130	15,388	77.05	2,971,909	1
Amar3	Target‐captured	501,968	100,998,343	66,952	83.25	14,062,457	5
Amar4	Target‐captured	202,284	68,522,780	35,876	77.68	7,715,135	20
Amar5	Target‐captured	385,463	87,449,366	43,939	81.81	9,412,478	4
Amar6	WGS	1,364,243	943,294,913	6165	23.13	1,427,133	34
Amar6	Target‐captured	701,154	318,305,473	5638	8.82	1,704,323	1
Amar7	Target‐captured	153,121	50,868,738	5920	40.96	1,534,621	0
Amar8	WGS	1,491,187	932,600,890	32,018	26.41	7,430,939	26
Amar9	WGS	92,940	28,540,424	145	23.28	42,555	0
Amar10	WGS	1,235,085	815,293,675	48,250	22.49	11,724,838	36
Amar11	WGS	417,692	272,969,227	3334	5.12	972,731	0
Archivesica nanshaensis*							
Anan1*	WGS	839,188	439,233,241	9953	19.63	3,468,497	12
Anan2*	WGS	374,882	216,389,232				
Anan3*	WGS	413,335	267,064,958				
*Archivesica okutanii*							
Aoku1	WGS	1340,943	667,236,497	3993	4.20	1,202,895	0
Aoku2	WGS	941,121	494,538,642	403,175	30.21	115,323,874	37^#^

For mitochondrial assemblies, reference‐guided assembly was unsuccessful for shell DNA due to few mitochondrial reads. Merged assemblies (*de novo* + reference‐guided) recovered complete sets of 37 mitochondrial genes (13 protein‐coding genes, 2 rRNA and 22 tRNA) in all fresh *A. marissinica* shells from WGS, whereas UCE‐captured datasets yielded only partial mt assemblies (1–20 gene fragments) (Tables 5, S8 and S9). Most ancient shells recovered fewer mt gene fragments than fresh shells (Tables 5, S8 and S9)—for example, specimens Amar8 (26 gene fragments) and Amar11 (none) in *A. marissinica* both from Haima seep in the South China Sea, three *A. nanshaensis* shells Anan1–3 (12 gene fragments) from an inactive northern South China Sea seep, and *A. okutanii* Aoku1 (none) from an Okinawa Trough vent. Notably, *A. marissinica* (Amar6 and Amar10) from Haima and *A. okutanii* Aoku2 from the Sagami Bay seep were preserved in better conditions, yielding near‐complete mitochondrial genomes (Tables 5, S8 and S9).

### 3.3 DNA Damage in Deep‐Sea Shells

Nucleotide misincorporation patterns were analysed with PMDtools (Figure 2h,i and Figures S8–S18). Endogenous reads mostly exceeded 100 bp (Figures S13–S18), but short reads (< 100 bp) were enriched after target‐capture enrichment (Figures S17–S18). Fresh shells showed minimal terminal damage (maximum 1.24% C‐to‐T misincorporation at the 5′ end) in both WGS and UCE‐captured datasets (Figures 2h,i, S8 and S10). Ancient shell DNA exhibited elevated damage levels (Figures 2h,i, S9, S11 and S12; Table S2), on average 3.46% or 15.97% C‐to‐T misincorporation at the 5′ end using bwa‐mem2‐mapped and bbmap‐mapped reads, respectively.

### 3.4 Phylogenetic Relationships Based on UCEs and Mitochondrial Genes

There were 0–230 UCEs recovered from WGS data and 142–1001 UCEs from UCE‐captured data (Table S10). Published vesicomyid datasets yielded 28 (*Abyssogena phaseoliformis*) to 1361 (*Calyptogena fausta*) UCEs (Table S10). After excluding taxa with insufficient loci, three UCE matrices were analysed (Tables S4 and S5): *Matrix‐A* and *Matrix‐B* (v1 probe set) and *Matrix‐C* (v2 probe set with additional transcriptome data).

**FIGURE 3:**
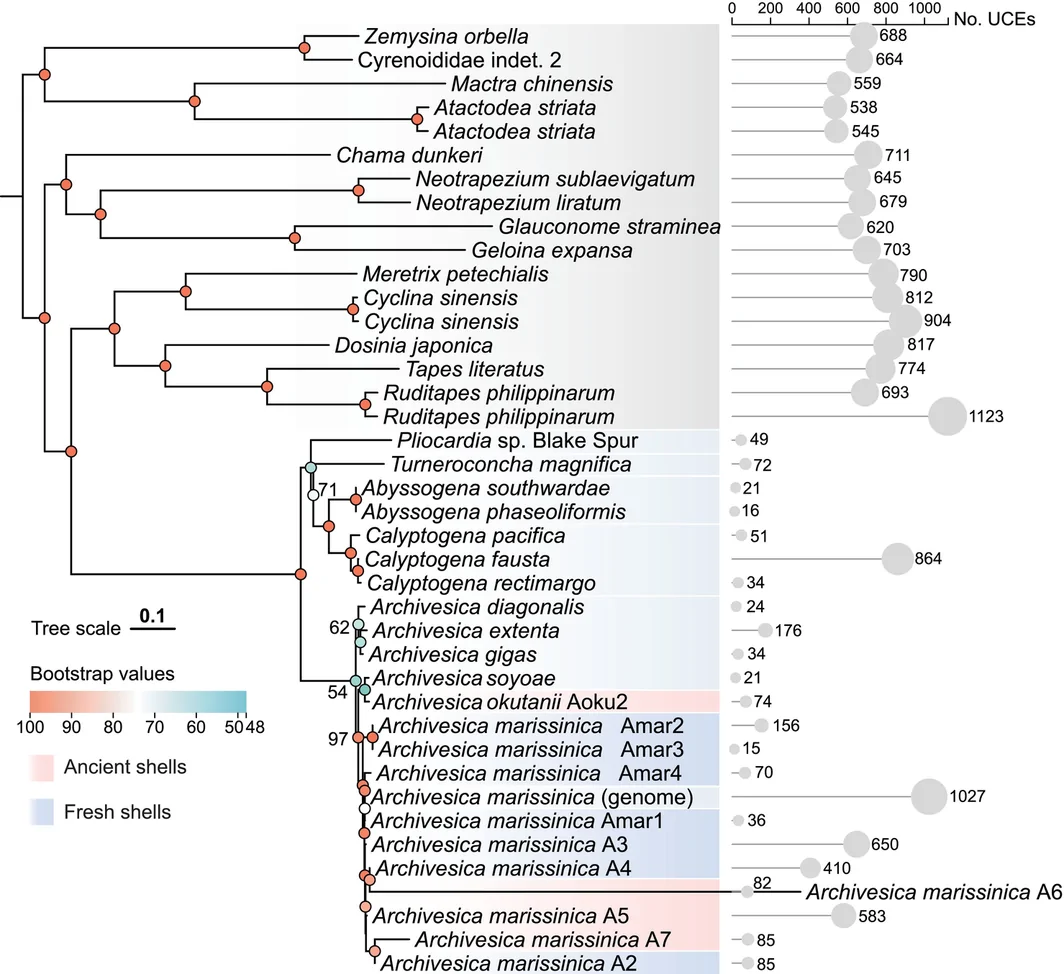
Phylogenetic tree of Venerida reconstructed by maximum likelihood using IQTREE2 with the UCE matrix *Matrix‐A* (1,246 alignments). Outgroups outside of Vesicomyidae, grey block; non‐shell data, light blue blocks; fresh‐shell data, blue blocks; ancient‐shell data, pink blocks.

Including the available outgroup venerid data from Li et al. (2025), ML topology of *Matrix‐A* with 1246 partitions (Figure 3) recovered well‐supported family‐level relationships in Venerida (ultrafast bootstrap = 100) and two major clades within the vesicomyid subfamily Pliocardiinae that contains genus *Archivesica* (ultrafast bootstrap = 100): (*Pliocardia* + (*Turneroconcha* + (*Abyssogena* + *Calyptogena*))) and a distinct *Archivesica* clade (((*Archivesica okutanii* + *Archivesica soyoae*) + *Archivesica marissinica* group) + (*Archivesica gigas* + other *Archivesica* species)). *Matrix‐B* (1,036 partitions) supported the same topology with improved support among genera (Figure S19), such as (*Pliocardia* sp. Blake Spur + (*Turneroconcha magnifica* + (*Abyssogena* + *Calyptogena*))) with ultrafast bootstrap ≥ 97. Within *Archivesica*, three subclades received strong support (ultrafast bootstrap = 97), with (*Archivesica okutanii* + *Archivesica soyoae*) sister to the *Archivesica marissinica* group (ultrafast bootstrap 100). However, the placement of *Archivesica extenta* was uncertain (ultrafast bootstrap = 59/80 in *Matrix‐A*/*Matrix‐B*). With more transcriptome data, *Matrix‐C* (488 partitions) using transcriptome‐derived probes yielded a conflicting and weakly supported topology of Pliocardiinae (Figure S20), with *Pliocardia* sp. Blake Spur placed as the basal species (ultrafast bootstrap = 50) and *Archivesica* in a bipartition with other pliocardiine species with low support (ultrafast bootstrap = 28). Nevertheless, shell‐derived *A. marissinica* UCEs consistently formed a clade (ultrafast bootstrap = 97–100) (Figures 3, S19 and S20).

**FIGURE 4:**
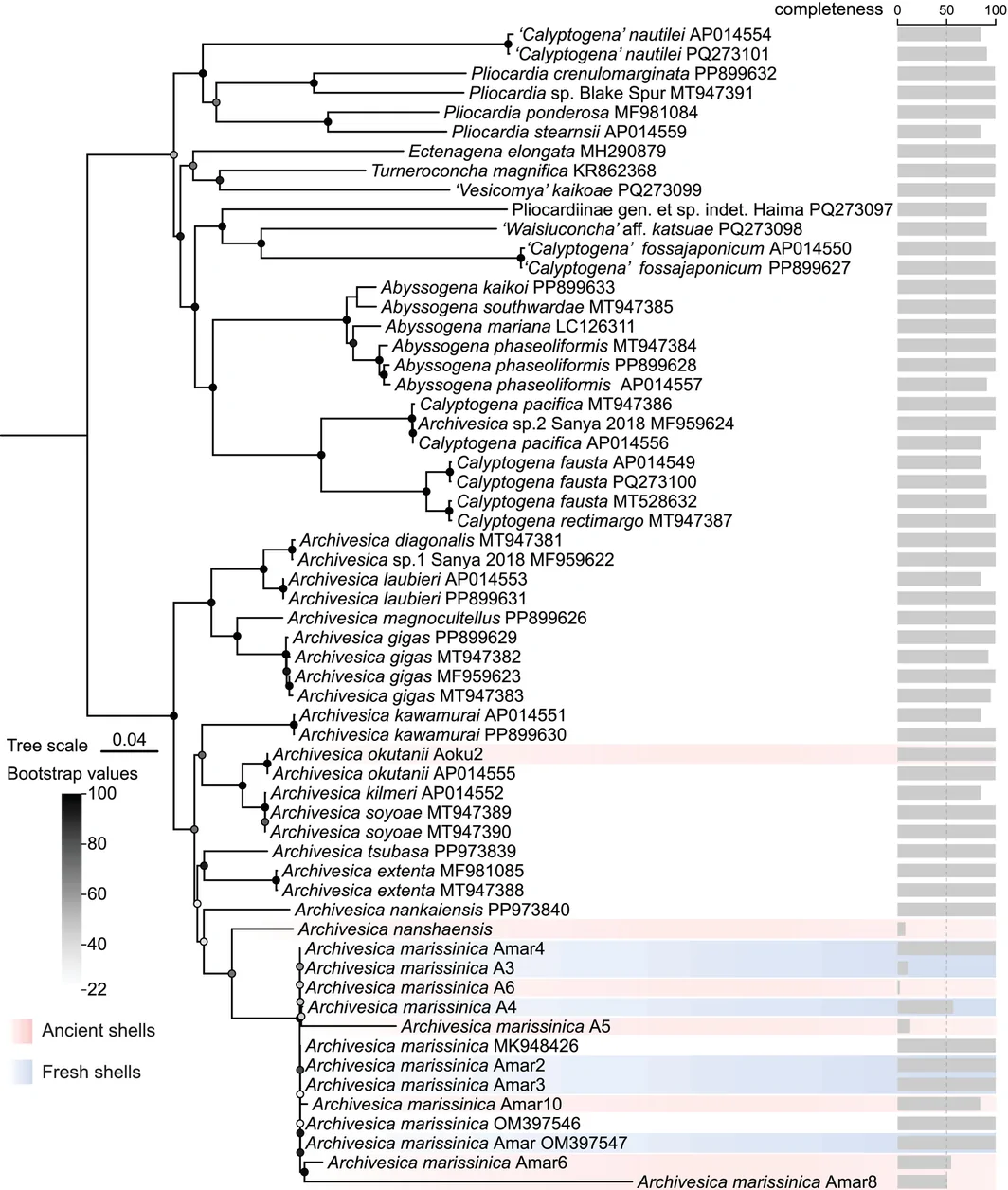
Phylogenetic tree of Pliocardiinae reconstructed by maximum likelihood using IQ‐TREE2 using concatenated 13 mitochondrial PCGs. Completeness, base completeness in the concatenated *Matrix‐D* (11,441 bp, details in Table S5). Information after species name, GenBank accession number or specimen ID.

We further reconstructed phylogenetic relationships within Pliocardiinae using *Matrix‐D* (11,441 bp, Figure 4), which included 13 concatenated PCGs of available mitochondrial genomes from 31 species (Table S6). A two‐clade topology was recovered, similar to *Matrix‐A* (Figure 3). Mitochondrial shell DNA of the three *Archivesica* species clustered robustly with fresh soft tissue data from conspecific or sister species, validating their taxonomic assignments. However, some subclade placements differed from UCE topologies (Figures 3, 4, S19 and S20), including the position of (*Ectenagena elongata* + (*Turneroconcha magnifica* + *‘Vesicomya’ kaikoae*)) outside (*‘Calyptogena’ nautilei* + *Pliocardia*) and weak support for *Archivesica kawamurai*, (*Archivesica tsubasa* + *Archiveisca extenta*) and *Archivesica nankaiensis* (ultrafast bootstrap = 31–74).

## 4 Discussion

Targeted enrichment strategies have been widely applied to vertebrate aDNA studies (Zavala et al. 2022; Salem et al. 2025), but tailored schemes for non‐vertebrate taxa remain underdeveloped. UCEs have transformed phylogenomics in contemporary taxa and show promise for degraded materials, yet most studies to date have largely focused on fresh and historical museum specimens rather than ancient biominerals (Derkarabetian et al. 2019; Girard and Chovanec 2025). Here, we extend UCE capture to ancient deep‐sea vesicomyid shells using the Bivalve UCE 2k v.1 probe set, and demonstrate that efficient recovery of nuclear loci enables phylogenomics from highly degraded calcium carbonate biominerals.

### 4.1 Performance of UCE Target Capture for Shell aDNA

Across different tissue types (fresh soft tissue, fresh shells, ancient shell ~1700–1100 years BP; Table 1), mapping success differed markedly between mitochondrial and nuclear fractions, ranging from 20 to 39,281 mitochondrial reads and from 403 thousand to 29 million nuclear reads (Table 2 and Figure 2a,b). The relative lack of mitochondrial reads and contigs in UCE capture datasets (Tables 2 and 5) likely reflected the additional enrichment step during library preparation, which selectively retains nuclear reads complementary to the UCE probes and washes away non‐target molecules, including most mitochondrial molecules. Ancient shells of *Archivesica marissinica* and *A. okutanii* produced an average of 5.58% mapped reads (4,083,431 nuclear and 598 mitochondrial on average, Table 3), lower than 7.13% endogenous fractions reported for ~100–7000 years BP (before present) *Mytilus* mussel shells (Martin‐Roy et al. 2024) but higher than yields from ~1000–7000 years BP gastropod shells (< 20 mitochondrial reads, < 10,000 nuclear reads per library) (Sullivan et al. 2021).

Our results unequivocally demonstrated that UCE capture substantially enhanced the yield of informative nuclear reads and loci. Non‐captured fresh and ancient *A. marissinica* shells had low on‐target reads (0.67% and 0.08%), compared with in fresh soft tissue (16.65%). UCE capture of fresh *A. marissinica* shells yielded 25.42% on‐target reads and 595 UCEs on average, compared with only 0.67% on‐target reads and 127 UCEs from WGS data (Tables 4 and S10). Target capture of ancient shell (Amar6) yielded higher on‐target reads (from 0.01% to 0.45%) and UCEs (from 12 to 145) (Tables 4 and S10); the same pattern occurred in two shells (Amar5 and Amar7) with DNA damage signals (Figure S9), reaching up to19.83% on‐target reads and 926 UCEs (Tables 4 and S10). Target capture substantially improved on‐target rates in ancient shell samples relative to WGS (median: 5.78% vs. 0.06%, *p* < 0.05, Table S7), highlighting the necessity of target enrichment strategies when working with ancient or degraded specimens. These values fall within expected ranges for target capture on degraded materials and underscore the efficacy of UCE enrichment for shell aDNA compared to WGS, despite inherently lower endogenous content and higher contamination compared to bones and soft tissues. For instance, WGS of ancient remains (Carpenter et al. 2013; Enk et al. 2014; Suchan et al. 2022; Martin‐Roy et al. 2024) resulted in an average of ~0.09%–35% reads mapping to the nuclear genome (*Mytilus* shells, mammal bones and teeth, etc.), whereas target capture of mammal substrates from thousands to over 10,000 years BP reached ~4%–80%. UCE capture studies targeting fresh and historical collections (2–180 years) yielded roughly 0%–36.7% of on‐target reads and 20%–92% targeted UCEs (Blaimer et al. 2016; McCormack et al. 2016; Roycroft et al. 2022).

Unlike bones or soft tissues, mollusc shells are calcium carbonate‐dominated, low‐cellularity substrates in which endogenous DNA is only secondarily entrapped, resulting in overall low endogenous content (~0.09%–30%), high contamination and complex microstructure (Der Sarkissian et al. 2020; Ferreira et al. 2020; Martin‐Roy et al. 2024). Consequently, even with the same UCE capture strategy, mapped‐read fractions from shells are expected to be lower than those from historical or ancient mammalian materials. The high on‐target rates and proportion of recovered UCEs in captured shells (e.g., Amar3, Amar4) suggest that probe‐based enrichment increases the fraction of target molecules recovered from complex libraries and highlight the potential of this approach for population and phylogenomic studies as more datasets become available.

### 4.2 Shell aDNA Damage, Sample Preservation and Environmental Effects

Although our ancient samples showed reduced sequence lengths compared to tissues and fresh shells, consistent with DNA degradation, most reads exceeded 100 bp (Figures S17–18), unlike the highly fragmented aDNA reported in other studies (Briggs et al. 2007; Maricic et al. 2010; Gansauge et al. 2017; Dalal et al. 2023). The relatively long read‐length distribution of our data may partly reflect the cleanup step in the library preparation kit, which preferentially removed the shortest fragments. Short fragments were also enriched in fresh‐shell libraries (Figures S17 and S18), indicating that length profiles could reflect both DNA degradation and library/capture‐related biases rather than a simple ancient‐versus‐modern contrast. Although extraction blanks were included during DNA extraction, they were not sequenced in the present study. We acknowledge this as a limitation and note that sequencing blank controls in future work would provide a more direct assessment of contamination.

Authenticity of aDNA rests on postmortem damage signatures (Sawyer et al. 2012), particularly elevated 5′ C‐to‐T substitution. Reported damage levels for shallow‐water shell aDNA vary with shell structure and preservation: 1%–25% in archaeological shells (~1000 years BP), 15%–30% in palaeontological shells (~7000 years BP); and < 5% in modern fresh and discarded shells (Sullivan et al. 2021; Psonis et al. 2022; Martin‐Roy et al. 2024). Bivalve shells in permafrost for up to 100,000 years showed low deamination levels (3.9%–9.4% C‐to‐T), reflecting that cold, stable conditions can slow DNA damage (Der Sarkissian et al. 2020). Herein, we observed a range of C‐to‐T misincorporation signals for ancient *A. marissinica* shells (Figures S9 and S11, 0.48%–5.65%, 8.88%–17.63%) using two mappers, irrespective of sequencing approach (WGS or UCE capture). The detected signal levels are close to the known archaeological shells ~1000 years BP (Sullivan et al. 2021). Instead, fresh *A. marissinica* shells exhibited minimal damage (0.24%–1.24% C‐to‐T misincorporation). Regarding the heterogeneity between bbmap‐ and bwa‐mem2‐mapped reads (Table 2), a varied range of damage signals was observed (Figure 2h,i). Our results indicated that mapping metrics may be influenced by sample preservation, library cleanup/bead ratio, reference choice and mapper choice, therefore affecting the DNA damage estimation. The damage signals from our ancient shells support a general association between deamination and time since death (Sawyer et al. 2012; Kistler et al. 2017) and suggest that combining damage metrics with independent dating could enable relative age estimation for shell assemblages.

Our shells come from deep‐sea habitats with contrasting physicochemical regimes: hydrocarbon seeps (*A. marissinica* and *A. okutanii*) and a hydrothermal vent (*A. okutanii*). Bottom waters at seeps are typically < 4°C (Ke et al. 2022), whereas vent waters can be substantially warmer (Nakamura et al. 2015; Watanabe and Kojima 2015). Geochemical profiles also differ among these habitats (Kawagucci 2015; Wang et al. 2018). These factors likely influence aDNA preservation via temperature, pH, redox state and exposure to reactive sulfur and metals. The vent shell (Aoku1, ~1200 years BP) showed a high deamination signal (35.65% C‐to‐T from bbmap‐mapped reads) and coincided with reduced endogenous yield (only 3993 nuclear contigs and no mitochondrial contigs, Table 5). We hypothesize that the distinct physicochemical conditions of vent environments may accelerate post‐mortem DNA degradation, in contrast to the colder and more stable deep‐sea conditions at seep sites, which appear more conducive to long‐term aDNA preservation. However, the present study is limited to a small number of specimens from a small set of sites, and the observed high mismatches may reflect bias related to reference quality (e.g., the *A. okutanii* transcripts). Consequently, an extrapolation to general habitat differences (such as vents versus seeps) should be avoided until it has been tested with larger sample sizes and improved reference genomes.

Additionally, the two relatively “younger” ancient shell specimens, Amar10 at 1282 years BP and Aoku2 at 1135 years BP, were less weathered than other older shells (Figures S2–S4, Table S1). This finding also demonstrates that the periostracum (the outer organic layer of the shell) can persist for long periods under the seafloor conditions (Ambrose et al. 2015; Leclaire et al. 2023; Martin‐Roy et al. 2024), representing another source of ancient DNA and protein information. Retaining a high proportion of periostracum was linked to better DNA preservation (Martin‐Roy et al. 2024), as evidenced by high on‐target UCE reads/loci numbers for Amar10 and Aoku2 (Tables 3, 4 and S9) and complete mitochondrial genomes recovered from them (Tables 5, S7 and S8). Our results indicate that deep‐sea habitats are not uniformly advantageous for aDNA preservation and that the exposure time of shells on the seafloor may also be a determinant of preservation. This underscores the preservation of periostracum as an indicator of DNA preservation potential and the importance of considering micro‐environmental geochemistry when prospecting for ancient biomolecules.

### 4.3 Phylogenetic Implications for Vesicomyid Clams

Our UCE‐based phylogenies, despite limited taxon sampling, provide new insights into the relationships within Pliocardiinae. The two UCE‐based *Matrix‐1 and Matrix‐2* recovered two main clades within Pliocardiinae (Figures 3, S13 and S14), consistent with prior hypotheses based on partial *cox1* sequences by He et al. (2023) and Audzijonyte et al. (2012). But they differed from earlier topologies (Decker et al. 2012; Johnson et al. 2017; Gao et al. 2025) inferred from *cox1* and three nuclear markers (*H3*, *18S* and *28S*) or transcriptome‐based datasets, which placed *Archivesica* differently, as sister to the subclade of *T. magnifica* and *‘Vesicomya’ kaikoae*. In our trees, *Archivesica* formed a distinct clade, with the Sagami Bay (Aoku2) *A. okutanii* shell sister to *A. soyoae* (ultrafast bootstrap = 100), consistent with previous results (Figure S13). All *A. marissinica* specimens formed a single, well‐supported clade, though ancient shells (Amar6, Amar7 and Amar10) exhibited long branches, likely attributing to post‐mortem DNA damage rather than true evolutionary divergence (Leonardi et al. 2017). Using the newly available transcriptome dataset, we also reconstructed another UCE tree using the v2 probe set, which was designed from bivalve transcriptomes. This analysis placed *Pliocardia* sp. Blake Spur as sister to all other pliocardiines and yielded a less strongly supported bipartition between *Archivesica* and the other pliocardiine species (Figure S14). Despite these differences among our UCE‐based topologies, all analyses consistently recover *Archivesica* monophyly, aligning with recent phylogenomic revisions (Gao et al. 2025).

Mitochondrial sequences remain more widely available for most non‐model bivalves, including vesicomyid clams (Gao et al. 2025). Our mitogenome tree supported two major clades consistent with their contrasting symbiont associations (*Candidatus* Vesicomyosocius vs. *Candidatus* Ruthia), congruent with UCE results (Figures 4, S13 and S14) and *cox1* results (Audzijonyte et al. 2012). Given the discordance between mitochondrial and nuclear markers and the complexity of vesicomyid diversification and colonization of deep‐sea chemosynthetic habitats, denser taxon sampling and higher‐quality genomic datasets are needed to stabilize genus‐level relationships within Pliocardiinae. Additionally, targeted mitogenome capture would complement UCE‐based nuclear phylogenies, allowing robust phylogenetic placement of poorly preserved or low‐coverage shell specimens when nuclear loci are sparse. Combined mitogenome capture and UCE enrichment should help integrate ancient shell material into broader phylogenomic analyses of molluscs for which such material is plentiful.

## 5 Conclusion

UCE target capture is an efficient strategy for recovering phylogenomic data from highly weathered, ancient deep‐sea vesicomyid shells. By increasing data recovery from low endogenous DNA content, this approach enables phylogenetic reconstruction and helps clarify how environmental and exposure time may influence shell DNA preservation. As a complement to existing genomic tools (Der Sarkissian et al. 2020; Li et al. 2025, 2026), the integration of target capture with shell aDNA will significantly advance our ability to reconstruct evolutionary histories, assess historical population connectivity and inform conservation strategies for marine bivalves and other calcifying organisms. This is crucial for deep‐sea ecosystems, where animals with shells such as bivalves act as key ecological engineers, but many species are only known from empty shells or very old museum material (Kiel and Little 2006; Kantor et al. 2020). The combination of UCEs and aDNA can turn subfossil and ancient shell material into a valuable resource for molecular ecology, opening up the possibility of extracting genetic information from museum specimens, archaeological molluscan shells and other shelled organisms.

## Author Contributions

Conceptualisation: J.‐W.Q, Y.‐X.L. Sampling: J.‐W.Q., C.C. Methodology: Y.‐X.L., Y.Z., Q.D., Y.Y., J.C.‐H.I. Analysis: Y.‐X.L. Writing – original draft: Y.‐X.L. Writing – review and editing: Y.‐X.L., J.‐W.Q., Y.Z., Q.D., C.C., C.T.S.L., M.Y., J.C.‐H.I., Y.Y. Funding acquisition: J.‐W.Q.

## Funding

This study was supported by the Research Grants Council of Hong Kong (General Research Fund: 12102222 & 12102125).

## Ethics Statement

All deep‐sea specimens were collected during these cruises under the relevant sampling permits issued by the cruise‐hosting institutions (Guangzhou Marine Geological Survey and JAMSTEC), in compliance with applicable national regulations and UNCLOS/marine scientific research requirements.

## Conflicts of Interest

The authors declare no conflicts of interest.

## Supporting information


**Table S1:** Results of carbon isotope analysis of *Archivesica marissinica* and *A. okutanii* shells. Cal year BP, calendar year before present. *, δ^13^C examined by IRMS, other specimens obtained δ^13^C_PDB_ by AMS.
**Table S2:** DNA damage profiles of deep‐sea shell data.
**Table S3:** Summary of vesicomyid clam assemblies using published data.
**Table S4:** Information for *Matrix‐A* and *Matrix‐B* and tree annotation using tvBOT. Tip_IDs, New_name, UCE, Italic_index, information used in tvBOT. rph, ama and csi, complete genome data (NCBI accession number: PRJNA479743, PRJNA629898 and PRJNA612143); #, assembled from published data (details in Table S2); *, data from NCBI Bioproject PRJNA1051799 (Li et al. 2025).
**Table S5:** Information for *Matrix‐C* and tree annotation using tvBOT. Tip_IDs, New_name, Italic_index, UCE, information used in tvBOT. rph, ama and csi, complete genome data (NCBI accession number: PRJNA479743, PRJNA629898 and PRJNA612143); #, assembled from published data (details in Table S2); *, data from NCBI Bioproject PRJNA1051799 (Li et al. 2025).
**Table S6:** Information for *Matrix‐D* and tree annotation using tvBOT. Tip_IDs, New_name, Italic_index, Completeness, information used in tvBOT. Completeness, base occurrence in concatenated alignment. Accession number after species name, GenBank accession number.
**Table S7:** Statistical comparisons of fresh shell vs. ancient shell and WGS vs. captured data (*Archivesica marissinica*) using two‐sided Mann–Whitney U test (Wilcoxon rank‐sum test). Mapped reads%, reads mapped to the nuclear genome and mitochondrial genome of *A. marissinica*.
**Table S8:** Mitochondrial contigs recovered from each specimen. Reference for BLASTn analysis, the mitochondrial genome of *Archivesica marissinica* (OM397546). #, target capture sequencing data; *, data from Li et al. (2023).
**Table S9:** Summary of mitochondrial contigs recovered from shells based on *de novo* and reference‐based assemblies. Assemblies blast against *Archivesica marissinica* (OM397546, 17,840 bp). id%, identity (%); Length; matched length (bp); q_start, start position of query sequence; q_end, end position of query sequence; ref_start, start position of reference sequence; ref_end, end position of reference sequence;e, e‐value; (p), partial gene.
**Table S10:** Number of UCEs captured from sequencing data. v1, UCEs match to v1 probe set; v2, UCEs match to v2 probe set.
**Figure S1:** Photos of fresh clam shells. a–d: Amar2, length = 9.7 cm; e–h: Amar3, length = 12.6 cm; i–m: Amar4, length = 14.1 cm. Left and right sides, two sides of the same valve.
**Figure S2:** Photos of ancient shells (*Archivesica marissinica*). a,b: Amar5, length = 11.3 cm; c,d: Amar6, length = 9 cm; e–h: Amar7, length = 5.5 cm. Left and right sides, two sides of the same valve.
**Figure S3:** Photos of ancient shells (*Archivesica marissinica*). a–d: Amar10, length = 10.7 cm; e–h: Amar11, length = 8.8 cm. Left and right sides, two sides of the same valve.
**Figure S4:** Photos of ancient shells (*Archivesica okutanii*). a–d: Aoku2, length = 11.7 cm; e–h: Aoku1, length = 12.6 cm. Left and right sides, two sides of the same valve.
**Figure S5:** Normalized reads similarity percentage between *Archivesica marissinica* samples based on reads mapped to the nuclear genome. WGS, Whole genome sequencing. Ama, tissue data from the reduced genome survey data of *A. marissinica* (randomly selected 10 Gb data from SRR11675938).
**Figure S6:** Sequencing depth of fresh shell reads in target UCE loci (*Archivesica marissinica*).
**Figure S7:** Sequencing depth of ancient shell reads in target UCE loci (*Archivesica marissinica*).
**Figure S8:** DNA damage analysis results for *Archivesica. marssinica* fresh shells using bbmap‐reads. No PMD score filtration performed.
**Figure S9:** DNA damage analysis results for *Archivesica marssinica* ancient shells using bbmap‐reads. PMD = 1 filtration performed.
**Figure S10:** DNA damage analysis results for *Archivesica marssinica* fresh shells using bwa‐mem2‐reads. No PMD score filtration performed.
**Figure S11:** DNA damage analysis results for *Archivesica marssinica* ancient shells using bwa‐mem2‐reads. No PMD score filtration performed.
**Figure S12:** DNA damage analysis results for *Archivesica okutanii* shells bbmap‐reads (a–d) and bwa‐mem2‐reads (e–i) using *A. okutanii* transcript as a reference. a,b: PMD = 1 filtration performed; c,d: PMD = 3 filtration performed; e,f: no filtration performed; g,h: PMD = 3 filtration performed.
**Figure S13:** BBmap‐reads length (log2) distribution of fresh and ancient *Archivesica marissnica* shells and *A. okutanii* shells (Aoku1 and Aoku2).
**Figure S14:** Bwa‐mem2‐reads length (log2) distribution of fresh and ancient *Archivesica marissnica* shells and *A. okutanii* shells (Aoku1 and Aoku2).
**Figure S15:** Length fraction of bbmap‐mapped reads for DNA damage analysis. Aoku, *Archivesica okutanii*.
**Figure S16:** Length fraction of bwa‐mem2‐mapped reads for DNA damage analysis. Aoku, *Archivesica okutanii*.
**Figure S17:** Length fraction of bbmap‐mapped reads for DNA damage analysis without 150‐bp reads. Aoku, *Archivesica okutanii*.
**Figure S18:** Length fraction of bwa‐mem2‐mapped reads for DNA damage analysis without 150‐bp reads. Aoku, *Archivesica okutanii*.
**Figure S19:** Topology using UCE matrix *Matrix‐B* with 1036 alignments from 25 sequencing data. Clade with shell DNA data, pink block. Node support value, only presents key nodes.
**Figure S20:** Topology using UCE matrix *Matrix‐C* with 488 alignments from 43 sequencing data (UCE target capture sequencing, metagenomic sequencing, transcriptome sequencing). Clade with shell DNA data, pink block. Node support value, only present value < 70.

## Data Availability

The raw sequencing data were deposited in the National Center for Biotechnology Information Sequence Read Archive (BioProject PRJNA1423908). Mitochondrial contigs, alignments and codes used in this study were deposited in FigShare (https://doi.org/10.6084/m9.figshare.31292794).

## References

[men70199-bib-0001] Adkins, J. F. , S. Griffin , M. Kashgarian , et al. 2002. “Radiocarbon Dating of Deep‐Sea Corals.” Radiocarbon 44, no. 2: 567–580. 10.1017/S0033822200031921.

[men70199-bib-0002] Ambrose, W. G. , G. Panieri , A. Schneider , et al. 2015. “Bivalve Shell Horizons in Seafloor Pockmarks of the Last Glacial‐Interglacial Transition: A Thousand Years of Methane Emissions in the Arctic Ocean.” Geochemistry, Geophysics, Geosystems 16, no. 12: 4108–4129. 10.1002/2015GC005980.

[men70199-bib-0003] Armbrecht, L. H. , M. J. L. Coolen , F. Lejzerowicz , et al. 2019. “Ancient DNA From Marine Sediments: Precautions and Considerations for Seafloor Coring, Sample Handling and Data Generation.” Earth‐Science Reviews 196: 102887. 10.1016/J.EARSCIREV.2019.102887.

[men70199-bib-0004] Audzijonyte, A. , E. M. Krylova , H. Sahling , and R. C. Vrijenhoek . 2012. “Molecular Taxonomy Reveals Broad Trans‐Oceanic Distributions and High Species Diversity of Deep‐Sea Clams (Bivalvia: Vesicomyidae: Pliocardiinae) in Chemosynthetic Environments.” Systematics and Biodiversity 10, no. 4: 403–415. 10.1080/14772000.2012.744112.

[men70199-bib-0005] Batistão, A. R. , J. A. Audino , E. Frigyik , et al. 2025. “Ultraconserved Element‐Based Phylogenomics and Siphonal Traits Illuminate the Evolution of Tellinoidean Clams (Mollusca: Bivalvia: Tellinoidea).” Zoological Journal of the Linnean Society 205, no. 3: zlaf159. 10.1093/zoolinnean/zlaf159.

[men70199-bib-0006] Bau, M. , S. Balan , K. Schmidt , and A. Koschinsky . 2010. “Rare Earth Elements in Mussel Shells of the Mytilidae Family as Tracers for Hidden and Fossil High‐Temperature Hydrothermal Systems.” Earth and Planetary Science Letters 299, no. 3–4: 310–316. 10.1016/J.EPSL.2010.09.011.

[men70199-bib-0007] Blaimer, B. B. , M. W. Lloyd , W. X. Guillory , and S. G. Brady . 2016. “Sequence Capture and Phylogenetic Utility of Genomic Ultraconserved Elements Obtained From Pinned Insect Specimens.” PLoS One 11, no. 8: e0161531. 10.1371/journal.pone.0161531. 27556533 PMC4996520

[men70199-bib-0008] Bolger, A. M. , M. Lohse , and B. Usadel . 2014. “Trimmomatic: A Flexible Trimmer for Illumina Sequence Data.” Bioinformatics 30, no. 15: 2114–2120. 10.1093/BIOINFORMATICS/BTU170. 24695404 PMC4103590

[men70199-bib-0009] Branstetter, M. G. , J. T. Longino , P. S. Ward , and B. C. Faircloth . 2017. “Enriching the Ant Tree of Life: Enhanced UCE Bait Set for Genome‐Scale Phylogenetics of Ants and Other Hymenoptera.” Methods in Ecology and Evolution 8, no. 6: 768–776. 10.1111/2041-210X.12742.

[men70199-bib-0010] Briggs, A. W. , U. Stenzel , P. L. F. Johnson , et al. 2007. “Patterns of Damage in Genomic DNA Sequences From a Neandertal.” Proceedings of the National Academy of Sciences 104, no. 37: 14616–14621. 10.1073/pnas.0704665104. PMC1976210 17715061

[men70199-bib-0011] BroadInstitue . 2019. Picard Toolkit. GitHub Repository. http://broadinstitute.github.io/picard/.

[men70199-bib-0012] Brown, J. , M. Pirrung , and L. A. McCue . 2017. “FQC Dashboard: Integrates FastQC Results Into a Web‐Based, Interactive, and Extensible FASTQ Quality Control Tool.” Bioinformatics 33, no. 19: 3137–3139. 10.1093/BIOINFORMATICS/BTX373. 28605449 PMC5870778

[men70199-bib-0013] Buenaventura, E. 2021. “Museomics and Phylogenomics With Protein‐Encoding Ultraconserved Elements Illuminate the Evolution of Life History and Phallic Morphology of Flesh Flies (Diptera: Sarcophagidae).” BMC Ecology and Evolution 21: 70. 10.1186/s12862-021-01797-7. 33910519 PMC8082969

[men70199-bib-0014] Bushnell, B. 2014. “BBMap: A Fast, Accurate, Splice‐Aware Aligner.”

[men70199-bib-0015] Campos, P. F. , O. E. Craig , G. Turner‐Walker , E. Peacock , E. Willerslev , and M. T. P. Gilbert . 2012. “DNA in Ancient Bone – Where Is It Located and How Should We Extract It?” Annals of Anatomy ‐ Anatomischer Anzeiger, Special Issue: Ancient DNA 194, no. 1: 7–16. 10.1016/j.aanat.2011.07.003. 21855309

[men70199-bib-0016] Capella‐Gutiérrez, S. , J. M. Silla‐Martínez , and T. Gabaldón . 2009. “trimAl: A Tool for Automated Alignment Trimming in Large‐Scale Phylogenetic Analyses.” Bioinformatics 25, no. 15: 1972–1973. 10.1093/bioinformatics/btp348. 19505945 PMC2712344

[men70199-bib-0017] Carpenter, M. L. , J. D. Buenrostro , C. Valdiosera , et al. 2013. “Pulling Out the 1%: Whole‐Genome Capture for the Targeted Enrichment of Ancient DNA Sequencing Libraries.” American Journal of Human Genetics 93, no. 5: 852–864. 10.1016/j.ajhg.2013.10.002. 24568772 PMC3824117

[men70199-bib-0018] Chen, Z. , J. D. Gardner , Z. Sun , et al. 2025. “Ancient DNA From Shimao City Records Kinship Practices in Neolithic China.” Nature 648: 659–667. 10.1038/s41586-025-09799-x. 41299168 PMC12711557

[men70199-bib-0019] Chen, Z. , K. Vončina , and J. D. Sigwart . 2026. “Evaluating Probe Design for Phylogenomics Across Taxonomic Scales: First Steps for Applying Ultraconserved Elements in an Understudied Class (Mollusca: Polyplacophora).” Molecular Ecology Resources 26, no. 1: e70076. 10.1111/1755-0998.70076. 41230929 PMC12614043

[men70199-bib-0021] Dalal, V. , N. Pasupuleti , G. Chaubey , N. Rai , and V. Shinde . 2023. “Advancements and Challenges in Ancient DNA Research: Bridging the Global North–South Divide.” Genes 14, no. 2: 479. 10.3390/genes14020479. 36833406 PMC9956214

[men70199-bib-0022] Danecek, P. , J. K. Bonfield , J. Liddle , et al. 2021. “Twelve Years of SAMtools and BCFtools.” GigaScience 10, no. 2: 1–4. 10.1093/GIGASCIENCE/GIAB008. PMC7931819 33590861

[men70199-bib-0023] Decker, C. , K. Olu , R. L. Cunha , and S. Arnaud‐Haond . 2012. “Phylogeny and Diversification Patterns Among Vesicomyid Bivalves.” PLoS One 7, no. 4: e33359. 10.1371/JOURNAL.PONE.0033359. 22511920 PMC3325225

[men70199-bib-0024] Der Sarkissian, C. , P. Möller , C. A. Hofman , et al. 2020. “Unveiling the Ecological Applications of Ancient DNA From Mollusk Shells.” Frontiers in Ecology and Evolution 8: 37. 10.3389/fevo.2020.00037.

[men70199-bib-0025] Der Sarkissian, C. , V. Pichereau , C. Dupont , et al. 2017. “Ancient DNA Analysis Identifies Marine Mollusc Shells as New Metagenomic Archives of the Past.” Molecular Ecology Resources 17, no. 5: 835–853. 10.1111/1755-0998.12679. 28394451

[men70199-bib-0026] Derkarabetian, S. , L. R. Benavides , and G. Giribet . 2019. “Sequence Capture Phylogenomics of Historical Ethanol‐Preserved Museum Specimens: Unlocking the Rest of the Vault.” Molecular Ecology Resources 19, no. 6: 1531–1544. 10.1111/1755-0998.13072. 31448547

[men70199-bib-0027] Enk, J. M. , A. M. Devault , M. Kuch , Y. E. Murgha , J.‐M. Rouillard , and H. N. Poinar . 2014. “Ancient Whole Genome Enrichment Using Baits Built From Modern DNA.” Molecular Biology and Evolution 31, no. 5: 1292–1294. 10.1093/molbev/msu074. 24531081

[men70199-bib-0028] Faircloth, B. C. 2016. “PHYLUCE Is a Software Package for the Analysis of Conserved Genomic Loci.” Bioinformatics 32, no. 5: 786–788. 10.1093/BIOINFORMATICS/BTV646. 26530724

[men70199-bib-0029] Ferreira, S. , R. Ashby , G. J. Jeunen , et al. 2020. “DNA From Mollusc Shell: A Valuable and Underutilised Substrate for Genetic Analyses.” PeerJ 8: e9420. 10.7717/PEERJ.9420/SUPP-2. 32821530 PMC7396136

[men70199-bib-0030] Gansauge, M. T. , T. Gerber , I. Glocke , et al. 2017. “Single‐Stranded DNA Library Preparation From Highly Degraded DNA Using T4 DNA Ligase.” Nucleic Acids Research 45, no. 10: e79. 10.1093/NAR/GKX033. 28119419 PMC5449542

[men70199-bib-0031] Gao, K. , X. He , H. Wang , et al. 2025. “Phylogenomic Analyses of Pliocardiinae (Bivalvia: Vesicomyidae) Update Genus‐Level Taxonomy and Shed Light on Trait Evolution.” Cladistics 41, no. 4: 372–387. 10.1111/cla.70001. 40549446

[men70199-bib-0032] Geist, J. , H. Wunderlich , and R. Kuehn . 2008. “Use of Mollusc Shells for DNA‐Based Molecular Analyses.” Journal of Molluscan Studies 74, no. 4: 337–343. 10.1093/mollus/eyn025.

[men70199-bib-0033] Girard, M. G. , and K. R. Chovanec . 2025. “Unlocking the Genomic Potential of Historical and Formalin‐Fixed Specimens: Phylogenetic Insights From Museum‐Preserved Threadfin Fishes (Teleostei: Polynemidae).” PeerJ 13: e20029. 10.7717/peerj.20029. 42433661 PMC13353053

[men70199-bib-0034] González‐Delgado, S. , P. C. Rodríguez‐Flores , and G. Giribet . 2024. “Testing Ultraconserved Elements (UCEs) for Phylogenetic Inference Across Bivalves (Mollusca: Bivalvia).” Molecular Phylogenetics and Evolution 198: 108129. 10.1016/j.ympev.2024.108129. 38878989

[men70199-bib-0035] Goulding, T. C. , E. E. Strong , and A. M. Quattrini . 2023. “Target‐Capture Probes for Phylogenomics of the Caenogastropoda.” Molecular Ecology Resources 23, no. 6: 1372–1388. 10.1111/1755-0998.13793. 36997300

[men70199-bib-0036] Grabherr, M. G. , B. J. Haas , M. Yassour , et al. 2011. “Full‐Length Transcriptome Assembly From RNA‐Seq Data Without a Reference Genome.” Nature Biotechnology 29, no. 7: 644–652. 10.1038/nbt.1883. PMC3571712 21572440

[men70199-bib-0037] Green, E. J. , and C. F. Speller . 2017. “Novel Substrates as Sources of Ancient DNA: Prospects and Hurdles.” Genes 8, no. 7: 7. 10.3390/genes8070180. PMC5541313 28703741

[men70199-bib-0038] He, X. , T. Xu , C. Chen , et al. 2023. “Same (Sea) Bed Different Dreams: Biological Community Structure of the Haima Seep Reveals Distinct Biogeographic Affinities.” Innovation Geoscience 1, no. 2: 100019. 10.59717/J.XINN-GEO.2023.100019.

[men70199-bib-0086] Heaton, T. J. , P. Köhler , M. Butzin , et al. 2020. “Marine20—The Marine Radiocarbon Age Calibration Curve (0–55,000 cal BP).” Radiocarbon 62, no. 4: 779–820. 10.1017/rdc.2020.68.

[men70199-bib-0039] Ip, J. C.‐H. , T. Xu , J. Sun , et al. 2021. “Host–Endosymbiont Genome Integration in a Deep‐Sea Chemosymbiotic Clam.” Molecular Biology and Evolution 38, no. 38: 502–518. 10.1093/molbev/msaa241. 32956455 PMC7826175

[men70199-bib-0040] Johnson, S. B. , E. M. Krylova , A. Audzijonyte , H. Sahling , and R. C. Vrijenhoek . 2017. “Phylogeny and Origins of Chemosynthetic Vesicomyid Clams.” Systematics and Biodiversity 15, no. 4: 346–360. 10.1080/14772000.2016.1252438/SUPPL_FILE/TSAB_A_1252438_SM4913.EPS.

[men70199-bib-0041] Kádár, E. , and V. Costa . 2006. “First Report on the Micro‐Essential Metal Concentrations in Bivalve Shells From Deep‐Sea Hydrothermal Vents.” Journal of Sea Research 56, no. 1: 37–44. 10.1016/J.SEARES.2006.01.001.

[men70199-bib-0042] Kalyaanamoorthy, S. , B. Q. Minh , T. K. F. Wong , A. von Haeseler , and L. S. Jermiin . 2017. “ModelFinder: Fast Model Selection for Accurate Phylogenetic Estimates.” Nature Methods 14, no. 6: 587–589. 10.1038/nmeth.4285. 28481363 PMC5453245

[men70199-bib-0043] Kantor, Y. , N. Puillandre , and P. Bouchet . 2020. “The Challenge of Integrative Taxonomy of Rare, Deep‐Water Gastropods: The Genus *Exilia* (Neogastropoda: Turbinelloidea: Ptychatractidae).” Journal of Molluscan Studies 86, no. 2: 120–138. 10.1093/mollus/eyz037.

[men70199-bib-0044] Katoh, K. , and D. M. Standley . 2013. “MAFFT Multiple Sequence Alignment Software Version 7: Improvements in Performance and Usability.” Molecular Biology and Evolution 30, no. 4: 772–780. 10.1093/molbev/mst010. 23329690 PMC3603318

[men70199-bib-0045] Kawagucci, S. 2015. “Fluid Geochemistry of High‐Temperature Hydrothermal Fields in the Okinawa Trough.” In Subseafloor Biosphere Linked to Hydrothermal Systems: TAIGA Concept, edited by J. Ishibashi , K. Okino , and M. Sunamura , 387–403. Springer Japan. 10.1007/978-4-431-54865-2_30.

[men70199-bib-0046] Ke, Z. , R. Li , Y. Chen , et al. 2022. “A Preliminary Study of Macrofaunal Communities and Their Carbon and Nitrogen Stable Isotopes in the Haima Cold Seeps, South China Sea.” Deep Sea Research Part I: Oceanographic Research Papers 184: 103774. 10.1016/J.DSR.2022.103774.

[men70199-bib-0047] Keighley, X. , M. H. Bro‐Jørgensen , H. Ahlgren , et al. 2021. “Predicting Sample Success for Large‐Scale Ancient DNA Studies on Marine Mammals.” Molecular Ecology Resources 21, no. 4: 1149–1166. 10.1111/1755-0998.13331. 33463014 PMC8248401

[men70199-bib-0048] Kiel, S. , and C. T. S. Little . 2006. “Cold‐Seep Mollusks Are Older Than the General Marine Mollusk Fauna.” Science 313, no. 5792: 1429–1431. 10.1126/science.112628. 16960004

[men70199-bib-0049] Kirch, M. , A. Romundset , M. T. P. Gilbert , F. C. Jones , and A. D. Foote . 2021. “Ancient and Modern Stickleback Genomes Reveal the Demographic Constraints on Adaptation.” Current Biology 31, no. 9: 2027–2036.e8. 10.1016/j.cub.2021.02.027. 33705715

[men70199-bib-0050] Kistler, L. , R. Ware , O. Smith , M. Collins , and R. G. Allaby . 2017. “A New Model for Ancient DNA Decay Based on Paleogenomic Meta‐Analysis.” Nucleic Acids Research 45, no. 11: 6310–6320. 10.1093/nar/gkx361. 28486705 PMC5499742

[men70199-bib-0051] Leclaire, A. M. , E. N. Powell , R. Mann , and T. Redmond . 2023. “Taphonomic Indicators of Dead Ocean Quahog ( *Arctica islandica* ) Shell Age in the Death Assemblage of the Mid‐Atlantic Bight Continental Shelf.” PALAIOS 38, no. 7: 305–314. 10.2110/palo.2022.030.

[men70199-bib-0052] Leonardi, M. , P. Librado , C. Der Sarkissian , et al. 2017. “Evolutionary Patterns and Processes: Lessons From Ancient DNA.” Systematic Biology 66, no. 1: e1–e29. 10.1093/SYSBIO/SYW059. 28173586 PMC5410953

[men70199-bib-0053] Li, H. 2013. “Aligning Sequence Reads, Clone Sequences and Assembly Contigs With BWA‐MEM.” arXiv 1303: 3997‐1303.3997.

[men70199-bib-0055] Li, Y.‐X. , J. C.‐H. Ip , C. Chen , et al. 2025. “Phylogenomics of Bivalvia Using Ultraconserved Elements Reveal New Topologies for Pteriomorphia and Imparidentia.” Systematic Biology 74, no. 1: 16–33. 39283716 10.1093/sysbio/syae052

[men70199-bib-0056] Li, Y.‐X. , T. Xu , M. Perez , et al. 2026. “Sequencing Ultraconserved Elements (UCEs) for Marine Population Genomics: A Proof‐Of‐Concept Using a Deep‐Sea Mussel Species.” Evolutionary Applications 19, no. 1: e70195. 10.1111/eva.70195. 41536848 PMC12797253

[men70199-bib-0057] Li, Y.‐X. , Y. Zhang , J. C.‐H. Ip , et al. 2023. “Phylogenetic Context of a Deep‐Sea Clam (Bivalvia: Vesicomyidae) Revealed by DNA From 1 500‐Year‐Old Shells.” Zoological Research 44, no. 2: 353–356. 10.24272/j.issn.2095-8137.2022.404. 36866638 PMC10083220

[men70199-bib-0058] Maillet, N. , G. Collet , T. Vannier , D. Lavenier , and P. Peterlongo . 2014. “Commet: Comparing and Combining Multiple Metagenomic Datasets.” 2014 IEEE International Conference on Bioinformatics and Biomedicine (BIBM): 94–98. 10.1109/BIBM.2014.6999135.

[men70199-bib-0059] Maricic, T. , M. Whitten , and S. Pääbo . 2010. “Multiplexed DNA Sequence Capture of Mitochondrial Genomes Using PCR Products.” PLoS One 5, no. 11: e14004. 10.1371/journal.pone.0014004. 21103372 PMC2982832

[men70199-bib-0060] Marin, F. , and G. Luquet . 2004. “Molluscan Shell Proteins.” Comptes Rendus Palevol 3, no. 6: 469–492. 10.1016/j.crpv.2004.07.009.

[men70199-bib-0061] Markulin, K. , M. Peharda , R. Mertz‐Kraus , et al. 2019. “Trace and Minor Element Records in Aragonitic Bivalve Shells as Environmental Proxies.” Chemical Geology 507: 120–133. 10.1016/j.chemgeo.2019.01.008.

[men70199-bib-0062] Martin‐Roy, R. , J. Thyrring , X. Mata , et al. 2024. “Advancing Responsible Genomic Analyses of Ancient Mollusc Shells.” PLoS One 19, no. 5: e0302646. 10.1371/journal.pone.0302646. 38709766 PMC11073703

[men70199-bib-0063] McCormack, J. E. , W. L. E. Tsai , and B. C. Faircloth . 2016. “Sequence Capture of Ultraconserved Elements From Bird Museum Specimens.” Molecular Ecology Resources 16, no. 5: 1189–1203. 10.1111/1755-0998.12466. 26391430

[men70199-bib-0064] Minh, B. Q. , H. A. Schmidt , O. Chernomor , et al. 2020. “IQ‐TREE 2: New Models and Efficient Methods for Phylogenetic Inference in the Genomic Era.” Molecular Biology and Evolution 37, no. 5: 1530–1534. 10.1093/MOLBEV/MSAA015. 32011700 PMC7182206

[men70199-bib-0065] Nakamura, K. , S. Kawagucci , K. Kitada , H. Kumagai , K. Takai , and K. Okino . 2015. “Water Column Imaging With Multibeam Echo‐Sounding in the Mid‐Okinawa Trough: Implications for Distribution of Deep‐Sea Hydrothermal Vent Sites and the Cause of Acoustic Water Column Anomaly.” Geochemical Journal 49, no. 6: 579–596. 10.2343/geochemj.2.0387.

[men70199-bib-0066] Nurk, S. , A. Bankevich , D. Antipov , et al. 2013. “Assembling Genomes and Mini‐Metagenomes From Highly Chimeric Reads.” Lecture Notes in Computer Science (Including Subseries Lecture Notes in Artificial Intelligence and Lecture Notes in Bioinformatics) 7821: 158–170. 10.1007/978-3-642-37195-0_13.

[men70199-bib-0067] Psonis, N. , K. Vardinoyannis , and N. Poulakakis . 2022. “High‐Throughput Degraded DNA Sequencing of Subfossil Shells of a Critically Endangered Stenoendemic Land Snail in the Aegean.” Molecular Phylogenetics and Evolution 175: 107561. 10.1016/J.YMPEV.2022.107561. 35779768

[men70199-bib-0085] Ramsey, C. B. 2021. “OxCal v.4.4.4 [software].” https://c14.arch.ox.ac.uk/oxcal.html.

[men70199-bib-0068] Roycroft, E. , C. Moritz , K. C. Rowe , et al. 2022. “Sequence Capture From Historical Museum Specimens: Maximizing Value for Population and Phylogenomic Studies.” Frontiers in Ecology and Evolution 10: 931644. 10.3389/fevo.2022.931644.

[men70199-bib-0069] Salem, N. , M. S. van de Loosdrecht , A. P. Sümer , et al. 2025. “Ancient DNA From the Green Sahara Reveals Ancestral North African Lineage.” Nature 641, no. 8061: 144–150. 10.1038/s41586-025-08793-7. 40175549 PMC12043513

[men70199-bib-0070] Salter, J. F. , P. A. Hosner , W. L. E. Tsai , et al. 2022. “Historical Specimens and the Limits of Subspecies Phylogenomics in the New World Quails (Odontophoridae).” Molecular Phylogenetics and Evolution 175: 107559. 10.1016/j.ympev.2022.107559. 35803448

[men70199-bib-0071] Sawyer, S. , J. Krause , K. Guschanski , V. Savolainen , and S. Pääbo . 2012. “Temporal Patterns of Nucleotide Misincorporations and DNA Fragmentation in Ancient DNA.” PLoS One 7, no. 3: e34131. 10.1371/journal.pone.0034131. 22479540 PMC3316601

[men70199-bib-0073] Skoglund, P. , B. H. Northoff , M. V. Shunkov , et al. 2014. “Separating Endogenous Ancient DNA From Modern Day Contamination in a Siberian Neandertal.” Proceedings of the National Academy of Sciences of the United States of America 111, no. 6: 2229–2234. 10.1073/PNAS.1318934111/SUPPL_FILE/PNAS.201318934SI.PDF. 24469802 PMC3926038

[men70199-bib-0074] Stiller, J. , R. R. da Fonseca , M. E. Alfaro , B. C. Faircloth , N. G. Wilson , and G. W. Rouse . 2021. “Using Ultraconserved Elements to Track the Influence of Sea‐Level Change on Leafy Seadragon Populations.” Molecular Ecology 30, no. 6: 1364–1380. 10.1111/mec.15744. 33217068

[men70199-bib-0075] Suchan, T. , M. A. Kusliy , N. Khan , et al. 2022. “Performance and Automation of Ancient DNA Capture With RNA hyRAD Probes.” Molecular Ecology Resources 22, no. 3: 891–907. 10.1111/1755-0998.13518. 34582623 PMC9291508

[men70199-bib-0076] Sullivan, A. P. , S. Marciniak , A. O'Dea , T. A. Wake , and G. H. Perry . 2021. “Modern, Archaeological, and Paleontological DNA Analysis of a Human‐Harvested Marine Gastropod ( *Strombus pugilis* ) From Caribbean Panama.” Molecular Ecology Resources 21, no. 5: 1517–1528. 10.1111/1755-0998.13361. 33595921

[men70199-bib-0077] Walton, K. , L. Scarsbrook , K. J. Mitchell , et al. 2023. “Application of Palaeogenetic Techniques to Historic Mollusc Shells Reveals Phylogeographic Structure in a New Zealand Abalone.” Molecular Ecology Resources 23, no. 1: 118–130. 10.1111/1755-0998.13696. 35951485 PMC10087340

[men70199-bib-0078] Wang, X. , N. Li , D. Feng , et al. 2018. “Using Chemical Compositions of Sediments to Constrain Methane Seepage Dynamics: A Case Study From Haima Cold Seeps of the South China Sea.” Journal of Asian Earth Sciences, South China Sea Seep 168: 137–144. 10.1016/j.jseaes.2018.11.011.

[men70199-bib-0079] Wang, Y. , W. Li , Q. Li , Y. Zhou , Z. Gao , and D. Feng . 2021. “Deep‐Sea Carbonates Are a Reservoir of Fossil Microbes Previously Inhabiting Cold Seeps.” Frontiers in Marine Science 8: 698945. 10.3389/fmars.2021.698945.

[men70199-bib-0080] Watanabe, H. , and S. Kojima . 2015. “Vent Fauna in the Okinawa Trough.” In Subseafloor Biosphere Linked to Hydrothermal Systems: TAIGA Concept, edited by J. Ishibashi , K. Okino , and M. Sunamura , 449–459. Springer Japan. 10.1007/978-4-431-54865-2_34.

[men70199-bib-0081] Watanabe, H. , E. Seo , Y. Takahashi , et al. 2012. “Spatial Distribution of Sister Species of Vesicomyid Bivalves *Calyptogena okutanii* and *Calyptogena soyoae* Along an Environmental Gradient in Chemosynthetic Biological Communities in Japan.” Journal of Oceanography 69, no. 1: 129–134. 10.1007/S10872-012-0155-3.

[men70199-bib-0082] Wilkinson, L. 2011. “ggplot2: Elegant Graphics for Data Analysis by WICKHAM, H.” Biometrics 67, no. 2: 678–679. 10.1111/J.1541-0420.2011.01616.X.

[men70199-bib-0083] Xie, J. , Y. Chen , G. Cai , R. Cai , Z. Hu , and H. Wang . 2023. “Tree Visualization by One Table (tvBOT): A Web Application for Visualizing, Modifying and Annotating Phylogenetic Trees.” Nucleic Acids Research 1, no. 1256879: kad359. 10.1093/nar/gkad359. PMC10320113 37144476

[men70199-bib-0084] Zavala, E. I. , A. Aximu‐Petri , J. Richter , B. Nickel , B. Vernot , and M. Meyer . 2022. “Quantifying and Reducing Cross‐Contamination in Single‐ and Multiplex Hybridization Capture of Ancient DNA.” Molecular Ecology Resources 22, no. 6: 2196–2207. 10.1111/1755-0998.13607. 35263821

