# Supplementary material for: Target Capture of Ancient Shell DNA Enables Phylogenetic Reconstruction of Deep‐Sea Molluscs
Source: Mol Ecol Resour. 2026 Aug 30;26(7):e70199. doi: 10.1111/1755-0998.70199 (PMC13527289; doi:10.1111/1755-0998.70199)
Supplement: Supplementary file 1 [file MEN-26-e70199-s001.docx]

**Supplementary File 1: Tables and figures**

This document contains 10 supplementary tables and 20 supplementary figures.

**Table S1 Results of carbon isotope analysis of *Archivesica marissinica* and *A. okutanii* shells.** Cal yr BP, calendar year before present. *, δ^13^C examined by IRMS, other specimens obtained δ^13^C_PDB_ by AMS.

| Sample | δ^13^C ±  Err. 1δ (‰) | ^14^C age ± Err.  1δ (yr) | pMC ± Err.  1δ (%) | Δ^14^C ± Err. 1δ  (‰) | Calibrated year (Cal yr BP) | |
| --- | --- | --- | --- | --- | --- | --- |
|  |  |  |  |  | Mean | Range (95%) |
| *Archivesica marissinica* | | | | | | |
| Amar6* | 0.77±0.03 | 2,050±30 | 77.48±0.29 | -232.24±2.89 | 1,444 | 1,584–1,305 |
| Amar7* | 0.05±0.01 | 2,110±30 | 76.90±0.29 | -237.95±2.87 | 1,522 | 1,675–1,370 |
| Amar10 | 1.1±0.5 | 1,893±20 | 79.01±0.20 | -216.69±1.98 | 1,282 | 1,405–1,160 |
| Amar11 | -0.6±0.6 | 2,244±21 | 75.63±0.20 | -250.20±1.96 | 1,671 | 1,812–1,530 |
| *Archivesica okutanii* | | | | | | |
| Aoku1 | 0.2±1.2 | 1,815±24 | 79.78±0.24 | -209.02±2.38 | 1,200 | 1,326–1,074 |
| Aoku2 | -1.4±0.9 | 1,742±22 | 80.51±0.22 | -201.83±2.21 | 1,135 | 1,270–1,001 |

δ^13^C: carbon isotope ratio of carbonates, δ^13^C_PDB_ compared with standards used limestone called Pee Dee Belemnite; pMC: percent modern carbon, 100% pMC was defined at 1950; Δ^14^C indicates the carbon source.

**Table S2 DNA damage profiles of deep-sea shell data.**

| ID | mapper | shell status | C-T 5’ | G-A 3’ | pmd filtration |
| --- | --- | --- | --- | --- | --- |
| Amar1 | bbmap | fresh | 0.24% | 0.18% | 0 |
| Amar2 | bbmap | fresh | 1.04% | 0.31% | 0 |
| Amar3 | bbmap | fresh | 1.21% | 0.36% | 0 |
| Amar4 | bbmap | fresh | 1.24% | 0.30% | 0 |
| A2 | bbmap | fresh | 0.55% | 0.68% | 0 |
| A3 | bbmap | fresh | 0.38% | 0.76% | 0 |
| A4 | bbmap | fresh | 0.35% | 0.62% | 0 |
| Amar9 | bbmap | ancient | 16.67% | 6.67% | 1 |
| Amar6 | bbmap | ancient | 12.59% | 10.83% | 1 |
| Amar8 | bbmap | ancient | 16.99% | 8.24% | 1 |
| Amar10 | bbmap | ancient | 17.63% | 6.52% | 1 |
| Amar11 | bbmap | ancient | 13.89% | 7.14% | 1 |
| A5 | bbmap | ancient | 8.88% | 20.27% | 1 |
| A6 | bbmap | ancient | 13.37% | 18.86% | 1 |
| A7 | bbmap | ancient | 10.81% | 22.36% | 1 |
| Aoku1 | bbmap | ancient | 35.65% | 22.22% | 1 |
| Aoku2 | bbmap | ancient | 13.22% | 14.73% | 3 |
| Amar1 | bwa-mem2 | fresh | 0.25% | 1.03% | 0 |
| Amar2 | bwa-mem2 | fresh | 1.04% | 2.62% | 0 |
| Amar3 | bwa-mem2 | fresh | 1.23% | 9.40% | 0 |
| Amar4 | bwa-mem2 | fresh | 1.24% | 1.98% | 0 |
| A2 | bwa-mem2 | fresh | 0.54% | 1.02% | 0 |
| A3 | bwa-mem2 | fresh | 0.38% | 1.19% | 0 |
| A4 | bwa-mem2 | fresh | 0.37% | 1.43% | 0 |
| Amar9 | bwa-mem2 | ancient | 4.32% | 15.57% | 0 |
| Amar6 | bwa-mem2 | ancient | 1.03% | 15.10% | 0 |
| Amar8 | bwa-mem2 | ancient | 0.89% | 9.27% | 0 |
| Amar10 | bwa-mem2 | ancient | 1.64% | 5.90% | 0 |
| Amar11 | bwa-mem2 | ancient | 5.65% | 16.28% | 0 |
| A5 | bwa-mem2 | ancient | 0.48% | 1.45% | 0 |
| A6 | bwa-mem2 | ancient | 3.76% | 9.66% | 0 |
| A7 | bwa-mem2 | ancient | 0.57% | 2.30% | 0 |
| Aoku1 | bwa-mem2 | ancient | 4.72% | 12.39% | 0 |
| Aoku2 | bwa-mem2 | ancient | 11.56% | 16.84% | 3 |

**Table S3 Summary of vesicomyid clam assemblies using published data.**

| Species | Number of contigs | Total length | Min. length | Average length | Max. length | Data source | SRA number |
| --- | --- | --- | --- | --- | --- | --- | --- |
| *Abyssogena southwardae* | 39,922 | 18,970,149 | 128 | 475 | 11,576 | metagenomic data | SRR12077202 |
| *Archivesica extenta* | 569,233 | 134,654,232 | 78 | 237 | 200,407 | metagenomic data | SRR12077196 |
| *Calyptogena fausta* | 815,451 | 580,537,012 | 78 | 712 | 216,241 | genome skimming | SRR12008158 |
| *Calyptogena pacifica* | 61,649 | 32,318,021 | 128 | 524 | 15,219 | metagenomic data | SRR12077193 |
| *Calyptogena rectimargo* | 60,242 | 28,417,206 | 128 | 472 | 27,840 | metagenomic data | SRR12077201 |
| *Archivesica soyoae* | 49,944 | 22,812,861 | 128 | 457 | 19,372 | metagenomic data | SRR12077194 |
| *Turneroconcha magnifica* | 36,867 | 15,363,688 | 126 | 417 | 5,877 | transcriptome | SRR8217831 |
| *Pliocardia* sp. Blake Spur | 69,904 | 34,426,138 | 128 | 493 | 17,211 | metagenomic data | SRR12077197 |
| *Archivesica gigas* | 60,781 | 28,822,343 | 128 | 474 | 25,143 | metagenomic data | SRR12077198 |
| *Archivesica nanshaensis* | 2,913 | 968,596 | 81 | 333 | 3,938 | metagenomic data | SRR19046124, SRR19046125, SRR19046126 |
| *‘Calyptogena’ nautilei* | 201,778 | 141,103,252 | 182 | 699 | 27,444 | transcriptome | SRR29224622 |
| *Archivesica tsubasa* | 18,939 | 6,753,256 | 185 | 357 | 5,563 | transcriptome | SRR29224624 |
| *Archivesica magnocultellus* | 96,167 | 53,569,788 | 184 | 557 | 8,691 | transcriptome | SRR29224625 |
| *Archivesica nankaiensis* | 45,909 | 17,566,577 | 178 | 383 | 10,070 | transcriptome | SRR29224626 |
| *Archivesica laubieri* | 18,538 | 6,851,254 | 198 | 370 | 6,256 | transcriptome | SRR29224627 |
| *Archivesica kawamurai* | 104,317 | 62,839,956 | 180 | 602 | 16,702 | transcriptome | SRR29224628 |
| *Pliocardia crenulomarginata* | 36,787 | 14,308,442 | 190 | 389 | 5,357 | transcriptome | SRR29224630 |
| *‘Waisiuconcha’* aff. *katsuae* | 32,776 | 13,092,911 | 186 | 399 | 10,302 | transcriptome | SRR29224631 |
| *‘Vesicomya’ kaikoae* | 24,655 | 8,916,795 | 186 | 362 | 4,682 | transcriptome | SRR29224632 |
| *Abyssogena kaikoi* | 2,753 | 1,019,448 | 201 | 370 | 3,840 | transcriptome | SRR29224635 |
| Pliocardiinae gen. et sp. indet. Haima | 195,635 | 140,231,456 | 172 | 717 | 18,392 | transcriptome | SRR32094328 |

**Table S4 Information for *Matrix-A* and *Matrix-B* and tree annotation using tvBOT.** Tip_IDs, New_name, UCE, Italic_index, information used in tvBOT. rph, ama, and csi, complete genome data (NCBI accession number: PRJNA479743, PRJNA629898, and PRJNA612143); #, assembled from published data (details in Table S2); *, data from NCBI Bioproject PRJNA1051799 (Li et al. 2025).

| Tip_IDs | New_name | UCE | *Matrix-A* | *Matrix-B* | Italic_index |
| --- | --- | --- | --- | --- | --- |
| rph | *Ruditapes philippinarum* | 1,123 | yes | yes | 0,1 |
| ama | *Archivesica marissinica* | 1027 | yes | yes | 0,1 |
| Pliocardia_sp | *Pliocardia* sp. Blake Spur# | 49 | yes | yes | 0 |
| C_magnifica | *Turneroconcha magnifica* # | 72 | yes | yes | 0,1 |
| A_phaseoliformis | *Abyssogena phaseoliformis* # | 16 | yes | yes | 0,1 |
| A_southwardae | *Abyssogena southwardae* # | 21 | yes | yes | 0,1 |
| C_pacifica | *Calyptogena pacifica* # | 51 | yes | yes | 0,1 |
| C_fausta | *Calyptogena fausta* # | 864 | yes | yes | 0,1 |
| C_rectimargo | *Calyptogena rectimargo* # | 34 | yes | yes | 0,1 |
| A_diagonalis | *Archivesica diagonalis* # | 24 | yes | yes | 0,1 |
| C_extenta | *Archivesica extenta* # | 176 | yes | yes | 0,1 |
| A_gigas | *Archivesica gigas* # | 34 | yes | yes | 0,1 |
| P_soyoae | *Archivesica soyoae* # | 21 | yes | yes | 0,1 |
| Aoku2 | *Archivesica okutanii* Aoku2 | 74 | yes | yes | 0,1 |
| Amar1 | *Archivesica marissinica* Amar1 | 36 | yes | yes | 0,1 |
| Amar2 | *Archivesica marissinica* Amar2 | 156 | yes | yes | 0,1 |
| Amar3 | *Archivesica marissinica* Amar3 | 15 | yes | yes | 0,1 |
| Amar4 | *Archivesica marissinica* Amar4 | 70 | yes | yes | 0,1 |
| A2 | *Archivesica marissinica* A2 | 85 | yes | yes | 0,1 |
| A3 | *Archivesica marissinica* A3 | 650 | yes | yes | 0,1 |
| A4 | *Archivesica marissinica* A4 | 410 | yes | yes | 0,1 |
| A5 | *Archivesica marissinica* A5 | 583 | yes | yes | 0,1 |
| A6 | *Archivesica marissinica* A6 | 82 | yes | yes | 0,1 |
| A7 | *Archivesica marissinica* A7 | 85 | yes | yes | 0,1 |
| A10 | *Archivesica marissinica* A10 | 23 | no | yes | 0,1 |
| b004_psre | *Chama dunkeri* * | 711 | yes | no | 0,1 |
| b015_trli | *Neotrapezium liratum* * | 679 | yes | no | 0,1 |
| b065_atst | *Atactodea striata* * | 538 | yes | no | 0,1 |
| b066_nesu | *Neotrapezium sublaevigatum* * | 654 | yes | no | 0,1 |
| b067_rude | *Tapes literatus* * | 774 | yes | no | 0,1 |
| b069_mepe | *Meretrix petechialis* * | 790 | yes | no | 0,1 |
| b072_mact | *Mactra chinensis* * | 559 | yes | no | 0,1 |
| b073_cysi | *Cyclina sinensis* * | 812 | yes | no | 0,1 |
| b076_glch | *Glauconome straminea* * | 620 | yes | no | 0,1 |
| b078_doja | *Dosinia japonica* * | 817 | yes | no | 0,1 |
| b088_geex | *Geloina expansa* * | 703 | yes | no | 0,1 |
| b095_Mact | *Atactodea striata* * | 545 | yes | no | 0,1 |
| b096_unkn | Cyrenoididae indet. 2 * | 664 | yes | no |  |
| b107_dior | *Zemysina orbella* * | 688 | yes | no | 0,1 |
| b112_ruph | *Ruditapes philippinarum.* * | 693 | yes | no | 0,1 |
| csi | *Cyclina sinensis* | 904 | yes | no | 0,1 |

**Table S5 Information for *Matrix-C* and tree annotation using tvBOT.** Tip_IDs, New_name, Italic_index, UCE, information used in tvBOT. rph, ama, and csi, complete genome data (NCBI accession number: PRJNA479743, PRJNA629898, and PRJNA612143); #, assembled from published data (details in Table S2); *, data from NCBI Bioproject PRJNA1051799 (Li et al. 2025).

| Tip_IDs | New_name | Italic_index | No. UCEs |
| --- | --- | --- | --- |
| rph | *Ruditapes philipinum* | 0,1 | 163 |
| ama | *Archivesica marissinica* | 0,1 | 610 |
| Pliocardia_sp | *Pliocardia* sp. Blake Spur | 0 | 28 |
| C_magnifica | *Turneroconcha magnifica* | 0,1 | 83 |
| A_phaseoliformis | *Abyssogena phaseoliformis* | 0,1 | 12 |
| A_southwardae | *Abyssogena southwardae* | 0,1 | 12 |
| C_pacifica | *Calyptogena pacifica* | 0,1 | 19 |
| C_fausta | *Calyptogena fausta* | 0,1 | 497 |
| C_rectimargo | *Calyptogena rectimargo* | 0,1 | 19 |
| A_diagonalis | *Archivesica diagonalis* | 0,1 | 18 |
| C_extenta | *Archivesica extenta* | 0,1 | 88 |
| A_gigas | *Archivesica gigas* | 0,1 | 26 |
| P_soyoae | *Archivesica soyoae* | 0,1 | 18 |
| Aoku2 | *Archivesica okutanii* Aoku2 | 0,1 | 39 |
| Amar1 | *Archivesica marissinica* Amar1 | 0,1 | 16 |
| Amar2 | *Archivesica marissinica* Amar2 | 0,1 | 74 |
| Amar3 | *Archivesica marissinica* Amar3 | 0,1 | 15 |
| Amar4 | *Archivesica marissinica* Amar4 | 0,1 | 53 |
| A2 | *Archivesica marissinica* A2 | 0,1 | 57 |
| A3 | *Archivesica marissinica* A3 | 0,1 | 351 |
| A4 | *Archivesica marissinica* A4 | 0,1 | 234 |
| A5 | *Archivesica marissinica* A5 | 0,1 | 343 |
| A6 | *Archivesica marissinica* A6 | 0,1 | 63 |
| A7 | *Archivesica marissinica* A7 | 0,1 | 67 |
| A10 | *Archivesica marissinica* A10 | 0,1 | 28 |
| b004_psre | *Chama dunkeri* * | 0,1 | 405 |
| b015_trli | *Neotrapezium liratum* * | 0,1 | 377 |
| b065_atst | *Atactodea striata* * | 0,1 | 286 |
| b066_nesu | *Neotrapezium sublaevigatum* * | 0,1 | 358 |
| b067_rude | *Tapes literatus* * | 0,1 | 333 |
| b069_mepe | *Meretrix petechialis* * | 0,1 | 431 |
| b072_mact | *Mactra chinensis* * | 0,1 | 292 |
| b073_cysi | *Cyclina sinensis* * | 0,1 | 426 |
| b076_glch | *Glauconome straminea* * | 0,1 | 302 |
| b078_doja | *Dosinia japonica* * | 0,1 | 403 |
| b088_geex | *Geloina expansa* * | 0,1 | 384 |
| b095_Mact | *Atactodea striata* * | 0,1 | 307 |
| b096_unkn | Cyrenoididae indet. 2 * |  | 346 |
| b107_dior | *Zemysina orbella* * | 0,1 | 352 |
| b112_ruph | *Ruditapes philippinarum.* * | 0,1 | 349 |
| csi | *Cyclina sinensis* | 0,1 | 398 |
| ecnat | *‘Calyptogena’ nautilei* # | 0,1 | 318 |
| artst | *Archivesica* sp. Kumano # | 0,1 | 84 |
| arkut | *Archivesica nankaiensis* # | 0 | 233 |
| phnat | *Archivesica laubieri* # | 0,1 | 164 |
| eclat | *Archivesica kawamurai* # | 0,1 | 60 |
| arkat | *Archivesica tsubasa* # | 0,1 | 253 |
| plcrt | *Pliocardia crenulomarginata* # | 0,1 | 74 |
| plsat | *‘Waisiuconcha’* aff. *katsuae* # | 0 | 90 |
| iskat | *‘Vesicomya’ kaikoae* # | 0,1 | 34 |
| plhat | Pliocardiinae gen. et sp. indet. Haima # | 0 | 329 |

**Table S6 Information for *Matrix-D* and tree annotation using tvBOT.** Tip_IDs, New_name, Italic_index, Completeness, information used in tvBOT. Completeness, base occurrence in concatenated alignment. Accession number after species name, GenBank accession number.

| Tip_IDs | New_name | Italic_index | Completeness |
| --- | --- | --- | --- |
| Abyssogena_kaikoi_PP899633 | *Abyssogena kaikoi* PP899633 | 0,1 | 99.93 |
| Abyssogena_mariana_LC126311 | *Abyssogena mariana* LC126311 | 0,1 | 99.93 |
| Abyssogena_phaseoliformis__AP014557 | *Abyssogena phaseoliformis* AP014557 | 0,1 | 91.12 |
| Abyssogena_phaseoliformis_MT947384 | *Abyssogena phaseoliformis* MT947384 | 0,1 | 99.93 |
| Abyssogena_phaseoliformis_PP899628 | *Abyssogena phaseoliformis* PP899628 | 0,1 | 99.93 |
| Abyssogena_southwardae_MT947385 | *Abyssogena southwardae* MT947385 | 0,1 | 99.93 |
| Akebiconcha_kawamurai_AP014551 | *Archivesica kawamurai* AP014551 | 0,1 | 84.78 |
| Archivesica_diagonalis_MT947381 | *Archivesica diagonalis* MT947381 | 0,1 | 100 |
| Archivesica_gigas_MF959623 | *Archivesica gigas* MF959623 | 0,1 | 99.69 |
| Archivesica_gigas_MT947382 | *Archivesica gigas* MT947382 | 0,1 | 92.58 |
| Archivesica_gigas_MT947383 | *Archivesica gigas* MT947383 | 0,1 | 94.98 |
| Archivesica_gigas_PP899629 | *Archivesica gigas* PP899629 | 0,1 | 99.55 |
| Archivesica_kawamurai_PP899630 | *Archivesica kawamurai* PP899630 | 0,1 | 99.95 |
| Archivesica_marissinica_LAma1_N__718842 | *Archivesica marissinica* LAma1 | 0,1 | 100 |
| Archivesica_marissinica_LAma2_N__033805 | *Archivesica marissinica* LAma2 | 0,1 | 100 |
| Archivesica_marissinica_LAma3_N__563464 | *Archivesica marissinica* LAma3 | 0,1 | 100 |
| Archivesica_marissinica_MK948426 | *Archivesica marissinica* MK948426 | 0,1 | 100 |
| Archivesica_nanshaensis | *Archivesica nanshaensis* | 0,1 | 7.89 |
| Archivesica_okutanii_SB_NODE__132170 | *Archivesica okutanii* SB | 0,1 | 100 |
| Archivesica_sp__1_Sanya_2018_MF959622 | *Archivesica* sp. 1 Sanya 2018 MF959622 | 0 | 100 |
| Archivesica_sp__2_Sanya_2018_MF959624 | *Archivesica* sp. 2 Sanya 2018 MF959624 | 0 | 100 |
| Archivesica_sp__PP899626 | *Archivesica magnocultellus* PP899626 | 0 | 99.71 |
| Archivesica_tsubasa_PP973839 | *Archivesica tsubasa* PP973839 | 0,1 | 99.84 |
| Calyptogena_extenta_MF981085 | *Archivesica extenta* MF981085 | 0,1 | 99.92 |
| Calyptogena_extenta_MT947388 | *Archivesica extenta* MT947388 | 0,1 | 99.92 |
| Calyptogena_fausta_AP014549 | *Calyptogena fausta* AP014549 | 0,1 | 84.76 |
| Calyptogena_fausta_MT528632 | *Calyptogena fausta* MT528632 | 0,1 | 90.94 |
| Calyptogena_fausta_PQ273100 | *Calyptogena fausta* PQ273100 | 0,1 | 90.75 |
| Calyptogena_magnifica_KR862368 | *Turneroconcha magnifica* KR862368 | 0,1 | 99.83 |
| Calyptogena_pacifica_AP014556 | *Calyptogena pacifica* AP014556 | 0,1 | 84.78 |
| Calyptogena_pacifica_MT947386 | *Calyptogena pacifica* MT947386 | 0,1 | 100 |
| Calyptogena_rectimargo_MT947387 | *Calyptogena rectimargo* MT947387 | 0,1 | 99.91 |
| A5 | *Archivesica marissinica* A5 | 0,1 | 12.95 |
| Amar6 | *Archivesica marissinica* Amar6 | 0,1 | 54.6 |
| Amar8 | *Archivesica marissinica* Amar8 | 0,1 | 50.74 |
| Ectenagena_elongata_MH290879 | *Ectenagena elongata* MH290879 | 0,1 | 99.73 |
| Ectenagena_laubieri_AP014553 | *Archivesica laubieri* AP014553 | 0,1 | 84.78 |
| Ectenagena_laubieri_PP899631 | *Archivesica laubieri* PP899631 | 0,1 | 99.71 |
| Ectenagena_nautilei_AP014554 | *‘Calyptogena' nautilei* AP014554 | 0,1 | 84.67 |
| Ectenagena_nautilei_PQ273101 | *‘Calyptogena' nautilei* PQ273101 | 0,1 | 91.1 |
| Amar10 | *Archivesica marissinica* Amar10 | 0,1 | 84.38 |
| Isorropodon_fossajaponicum_AP014550 | *‘Calyptogena’ fossajaponica* AP014550 | 0,1 | 99.66 |
| Isorropodon_fossajaponicum_PP899627 | *‘Calyptogena’ fossajaponica* PP899627 | 0,1 | 99.66 |
| Isorropodon_kaikoae_PQ273099 | *‘Vesicomya’ kaikoae* PQ273099 | 0,1 | 99.32 |
| A3 | *Archivesica marissinica* A3 | 0,1 | 10.35 |
| A4 | *Archivesica marissinica* A4 | 0,1 | 56.83 |
| Phreagena_kilmeri_AP014552 | *Archivesica kilmeri* AP014552 | 0,1 | 84.78 |
| Phreagena_nankaiensis_PP973840 | *Archivesica nankaiensis* PP973840 | 0,1 | 100 |
| Phreagena_okutanii_AP014555 | *Archivesica okutanii* AP014555 | 0,1 | 99.79 |
| Phreagena_soyoae_MT947389 | *Archivesica soyoae* MT947389 | 0,1 | 100 |
| Phreagena_soyoae_MT947390 | *Archivesica soyoae* MT947390 | 0,1 | 100 |
| Pliocardia_crenulomarginata_PP899632 | *Pliocardia crenulomarginata* PP899632 | 0,1 | 99.41 |
| Pliocardia_ponderosa_MF981084 | *Pliocardia ponderosa* MF981084 | 0,1 | 99.72 |
| Pliocardia_sp__A3710_17_S18_MT947391 | *Pliocardia* sp. Blake Spur MT947391 | 0 | 99.69 |
| Pliocardia_stearnsii_AP014559 | *Pliocardia stearnsii* AP014559 | 0,1 | 84.56 |
| Pliocardiinae_sp__Haima_PQ273097 | Pliocardiinae gen. et sp. indet. Haima PQ273097 | | 90.93 |
| Vesicomya_aff__katsuae_PQ273098 | *‘Waisiuconcha'* aff*. katsuae* PQ273098 | 0,2 | 90.89 |
| A6 | *Archivesica marissinica* A6 | 0,1 | 2.38 |
| Archivesica_marissinica_OM397546 | *Archivesica marissinica* OM397546 | 0,1 | 100 |
| Archivesica_marissinica_OM397547 | *Archivesica marissinica* OM397547 | 0,1 | 100 |

**Table S7 Statistical comparisons of fresh shell *vs.* ancient shell and WGS *vs.* captured data (*A. marissinica*) using two-sided Mann-Whitney U test (Wilcoxon rank-sum test).** Mapped reads%, reads mapped to the nuclear genome and mitochondrial genome of *A. marissinica*.

| Comparison | Comparative groups | Median | *p* value | Standard effective size |
| --- | --- | --- | --- | --- |
| Mapped reads% | Fresh shell WGS | 9.2492 | 0.032 | 0.69 |
|  | Ancient shell WGS | 3.1823 |  |  |
|  | WGS | 3.7501 | 0.008 | 0.65 |
|  | Target capture | 2.1418 |  |  |
| On-target UCE reads% | Fresh shell | 0.97 | 0.029 | 0.55 |
|  | Ancient shell | 0.165 |  |  |
|  | Fresh shell WGS | 0.76 | 0.015 | 0.78 |
|  | Ancent shell WGS | 0.06 |  |  |
|  | Ancent shell WGS | 0.06 | 0.036 | 0.74 |
|  | Ancient shell target capture | 5.78 |  |  |
|  | WGS | 0.17 | 0.003 | 0.72 |
|  | Target capture | 20.14 |  |  |

**Table S8 Mitochondrial contigs recovered from each specimen.** Reference for BLASTn analysis, the mitochondrial genome of *Archivesica marissinica* (OM397546). #, target capture sequencing data; *, data from Li et al. (2023).

| Species | ID | Contigs | Genes | Coding genes+rRNA | Length (bp) |
| --- | --- | --- | --- | --- | --- |
| *Archivesica marissinica* | Amar1 | 1 | 37 | 15 | 16,601 |
|  | Amar2 | 1 | 37 | 15 | 16,662 |
|  | Amar3 | 1 | 37 | 15 | 16,702 |
|  | Amar4 | 1 | 37 | 15 | 17,384 |
|  | A2# | 1 | 1 | 1 | 275 |
|  | A3# | 4 | 5 | 5 | 2,954 |
|  | A4# | 11 | 20 | 14 | 10,201 |
|  | Amar9 | 0 | 0 | 0 | 0 |
|  | A5# | 3 | 4 | 4 | 1,513 |
|  | Amar6 | 18 | 34 | 15 | 12,584 |
|  | A6# | 2 | 1 | 1 | 467 |
|  | A7# | 0 | 0 | 0 | 0 |
|  | Amar8 | 29 | 26 | 14 | 12,087 |
|  | Amar10 | 9 | 36 | 15 | 16,707 |
|  | Amar11 | 0 | 0 | 0 | 0 |
| *Archivesica nanshaensis** | Anan* | 12 | 12 | 8 | 2,473 |
| *Archivesica okutanii* | Aoku2 | 1 | 37 | 15 | 17,549 |
|  | Aoku1 | 0 | 0 | 0 | 0 |

**Table S9 Summary of mitochondrial contigs recovered from shells based on *de novo* and reference-based assemblies.** Assemblies blast against *Archivesica marissinica* (OM397546, 17,840 bp). id%, identity (%); Length; matched length (bp); q_start, start position of query sequence; q_end, end position of query sequence; ref_start, start position of reference sequence; ref_end, end position of reference sequence;e, e-value; (p), partial gene.

| ID | No. | Sequences ID | Region | id% | Length | q_start | q_end | ref_start | ref_end | e-value |
| --- | --- | --- | --- | --- | --- | --- | --- | --- | --- | --- |
| Amar1 | 1 | OM397547 | complete 37 genes | 99.97 | 16457 | 1 | 16457 | 194 | 16648 | 0 |
| Amar2 | 1 | NODE_1507_length_16543_cov_16.477408 | complete 37 genes | 99.945 | 16370 | 3 | 16372 | 265 | 16631 | 0 |
| Amar3 | 1 | NODE_3631_length_16348_cov_64.571508 | non-coding | 99.969 | 16348 | 1 | 16348 | 16699 | 355 | 0 |
| Amar4 | 1 | NODE_3637_length_17384_cov_186.950199 | complete 37 genes | 99.927 | 16521 | 1 | 16519 | 237 | 16755 | 0 |
| A2 | 1 | NODE_19732_length_275_cov_1.106061 | cytb(p) | 100 | 275 | 1 | 275 | 8526 | 8800 | 5.65E-147 |
| A3 | 1 | NODE_1_length_1351_cov_5.018053 | cytb(p)-16s(p) | 99.9 | 1351 | 1 | 1351 | 9334 | 7984 | 0 |
|  | 2 | NODE_11157_length_898_cov_2.577345 | cox1(p) | 100 | 898 | 1 | 898 | 4786 | 5683 | 0 |
|  | 3 | NODE_40976_length_446_cov_3.680217 | ND6(p) | 99.776 | 446 | 1 | 446 | 552 | 996 | 0 |
|  | 4 | NODE_5_length_259_cov_0.802198 | 16s-atp8 | 100 | 259 | 1 | 259 | 9928 | 10186 | 1.13E-130 |
| A4 | 1 | NODE_2_length_1896_cov_3.066520 | ND6-ND4l-G-ND2(p) | 99.8 | 1903 | 1 | 1512 | 339 | 2240 | 0 |
|  | 2 | NODE_68029_length_288_cov_1.725118 | ND2(p)-D-T-12s(p) | 100 | 284 | 5 | 288 | 2251 | 2534 | 5.90E-152 |
|  | 3 | NODE_39078_length_400_cov_1.882353 | cox3(p) | 99.441 | 179 | 1 | 179 | 3699 | 3877 | 9.03E-92 |
|  | 4 | NODE_1725_length_2050_cov_5.686265 | cox3(p)-F-A-cox1(p) | 99.902 | 2050 | 1 | 2050 | 4256 | 6305 | 0 |
|  | 5 | NODE_32920_length_446_cov_2.563686 | cox2(p) | 100 | 446 | 1 | 446 | 6657 | 7102 | 0 |
|  | 6 | NODE_1488_length_2232_cov_20.341995 | cox2(p)-R-cytb-16s(p) | 99.373 | 2232 | 1 | 2232 | 7326 | 9557 | 0 |
|  | 7 | NODE_4_length_1331_cov_2.578150 | atp8(p)-ND4(p) | 99.96 | 1331 | 1 | 1331 | 10068 | 11398 | 0 |
|  | 8 | NODE_66541_length_290_cov_1.525822 | atp6(p) | 99.313 | 291 | 1 | 290 | 12065 | 12355 | 5.94E-152 |
|  | 9 | NODE_17268_length_677_cov_1.995000 | ND1(p) | 99.705 | 677 | 1 | 677 | 13400 | 14076 | 0 |
|  | 10 | NODE_127020_length_235_cov_0.924051 | ND5(p) | 99.574 | 235 | 1 | 235 | 14795 | 15029 | 3.83E-123 |
|  | 11 | NODE_22460_length_573_cov_1.467742 | ND5(p) | 100 | 573 | 1 | 573 | 15081 | 15653 | 0 |
| Amar6 | 1 | NODE_1354609_length_195_cov_24.593220 | non-coding | 100 | 195 | 1 | 195 | 1 | 195 | 1.16E-102 |
|  | 2 | NODE_608706_length_348_cov_3.487085 | ND6(p)-ND4L-G(p) | 99.713 | 348 | 1 | 348 | 865 | 1211 | 0 |
|  | 3 | NODE_16_length_306_cov_1.205240 | ND2(p) | 99.8 | 306 | 1 | 306 | 1820 | 2125 | 4.74E-155 |
|  | 4 | NODE_265420_length_614_cov_1.806331 | ND2(p)-D-T-12s(p) | 100 | 477 | 1 | 477 | 2148 | 2624 | 0 |
|  | 5 | NODE_273730_length_600_cov_1.818356 | 12s(p)-M-C-Y(p) | 100 | 600 | 1 | 600 | 2975 | 3574 | 0 |
|  | 6 | NODE_306486_length_552_cov_1.835789 | cox3(p)-F(p) | 99.273 | 550 | 3 | 552 | 3978 | 4527 | 0 |
|  | 7 | NODE_88318_length_1423_cov_2.424963 | F(p)-A-cox1(p) | 99.859 | 1423 | 1 | 1423 | 4534 | 5956 | 0 |
|  | 8 | NODE_163689_length_895_cov_2.045232 | cox1(p)-P-cox2(p) | 100 | 895 | 1 | 895 | 7278 | 6384 | 0 |
|  | 9 | NODE_189010_length_802_cov_2.086897 | cytb | 99.751 | 802 | 1 | 802 | 7519 | 8319 | 0 |
|  | 10 | NODE_176389_length_846_cov_2.710013 | 16s-Cytb | 100 | 846 | 1 | 846 | 8351 | 9196 | 0 |
|  | 11 | NODE_322599_length_532_cov_1.874725 | 16s(p)-atp8-ND4(p) | 100 | 532 | 1 | 532 | 9871 | 10402 | 0 |
|  | 12 | NODE_255652_length_632_cov_1.257658 | 16s(p) | 100 | 632 | 1 | 632 | 9880 | 9249 | 0 |
|  | 13 | NODE_269012_length_608_cov_1.576271 | ND4(p)-H(p) | 100 | 608 | 1 | 608 | 11022 | 11629 | 0 |
|  | 14 | NODE_46905_length_2296_cov_1.376746 | ATP6(p)-ND3-Q-I-K-L-ND1(p) | 99.949 | 1980 | 317 | 2296 | 11995 | 13974 | 0 |
|  | 15 | NODE_236451_length_671_cov_1.462963 | ND1(p)-V-N-ND5(p) | 99.851 | 671 | 1 | 671 | 13985 | 14655 | 0 |
|  | 16 | NODE_157719_length_920_cov_2.618031 | ND5(p)-L-W-noncoding | 100 | 920 | 1 | 920 | 15405 | 16324 | 0 |
|  | 17 | NODE_349626_length_503_cov_1.314554 | ND5(p) | 99.801 | 503 | 1 | 503 | 15405 | 14903 | 0 |
|  | 18 | NODE_765195_length_296_cov_1.301370 | non-coding | 100 | 296 | 1 | 296 | 16665 | 16370 | 1.30E-158 |
| Amar8 | 1 | NODE_1470901_length_195_cov_32.364407 | non-coding | 100 | 195 | 1 | 195 | 195 | 1 | 1.16E-102 |
|  | 2 | NODE_638260_length_309_cov_1.258621 | ND6(p) | 100 | 309 | 1 | 309 | 758 | 450 | 8.01E-166 |
|  | 3 | NODE_330074_length_455_cov_3.187831 | ND4L(p)G-ND2(p) | 99.78 | 455 | 1 | 455 | 1448 | 994 | 0 |
|  | 4 | NODE_7_length_519_cov_2.169683 | ND2(p) | 100 | 519 | 1 | 519 | 1833 | 2351 | 0 |
|  | 5 | NODE_666354_length_300_cov_0.982063 | ND2(p)-D-T-12s(p) | 100 | 300 | 1 | 300 | 2679 | 2380 | 7.85E-161 |
|  | 6 | NODE_223702_length_591_cov_2.192607 | 12s(p) | 100 | 591 | 1 | 591 | 3296 | 2706 | 0 |
|  | 7 | NODE_204831_length_631_cov_1.066787 | cox3(p) | 98.06 | 464 | 166 | 629 | 3909 | 4372 | 0 |
|  | 8 | NODE_15_length_399_cov_1.360248 | cox3(p)-F-A-cox1(p) | 100 | 399 | 1 | 399 | 4417 | 4815 | 0 |
|  | 9 | NODE_337661_length_449_cov_2.352151 | cox1(p) | 100 | 449 | 1 | 449 | 5034 | 5482 | 0 |
|  | 10 | NODE_1_length_751_cov_2.310089 | cox1(p) | 100 | 751 | 1 | 751 | 5511 | 6261 | 0 |
|  | 11 | NODE_137409_length_853_cov_1.743557 | cox1(p) | 100 | 567 | 1 | 567 | 6261 | 5695 | 0 |
|  | 12 | NODE_7_length_519_cov_2.169683 | cox1(p)-P-cox2(p) | 100 | 501 | 1 | 501 | 6518 | 7018 | 0 |
|  | 13 | NODE_1271228_length_244_cov_0.934132 | cox2(p) | 100 | 244 | 1 | 244 | 7300 | 7543 | 8.51E-130 |
|  | 14 | NODE_266922_length_522_cov_3.110112 | cytb(p) | 100 | 522 | 1 | 522 | 8424 | 7903 | 0 |
|  | 15 | NODE_5_length_554_cov_0.809224 | 16s(p) | 99.9 | 554 | 1 | 554 | 9306 | 9859 | 0 |
|  | 16 | NODE_379035_length_419_cov_2.043860 | atp8(p)-ND4(p) | 100 | 419 | 1 | 419 | 9853 | 10271 | 0 |
|  | 17 | NODE_245344_length_554_cov_0.916143 | 16s(p) | 100 | 554 | 1 | 554 | 9859 | 9306 | 0 |
|  | 18 | NODE_1169887_length_252_cov_0.834286 | ND4(p) | 99.603 | 252 | 1 | 252 | 10430 | 10681 | 1.46E-132 |
|  | 19 | NODE_189182_length_669_cov_1.711149 | ND4(p) | 99.766 | 428 | 5 | 432 | 11025 | 11452 | 0 |
|  | 20 | NODE_3_length_597_cov_3.905769 | E(p)-S-atp6(p) | 100 | 597 | 1 | 597 | 11709 | 12305 | 0 |
|  | 21 | NODE_21_length_295_cov_1.004587 | non-coding | 100 | 295 | 1 | 295 | 12308 | 12602 | 1.27E-150 |
|  | 22 | NODE_345118_length_443_cov_2.120219 | L-ND1(p) | 100 | 124 | 320 | 443 | 13149 | 13272 | 8.10E-63 |
|  | 23 | NODE_18_length_316_cov_2.748954 | ND1(p) | 100 | 316 | 1 | 316 | 13482 | 13797 | 2.91E-162 |
|  | 24 | NODE_556627_length_337_cov_1.165385 | V-N-ND5(p) | 100 | 337 | 1 | 337 | 14131 | 14467 | 0 |
|  | 25 | NODE_214156_length_610_cov_2.283302 | ND5(p) | 99.836 | 610 | 1 | 610 | 14769 | 15378 | 0 |
|  | 26 | NODE_14_length_415_cov_1.849112 | ND5(p) | 99 | 415 | 1 | 415 | 15454 | 15868 | 0 |
|  | 27 | NODE_16_length_388_cov_1.848875 | non-coding | 99.7 | 390 | 1 | 390 | 16270 | 16659 | 0 |
|  | 28 | NODE_723779_length_288_cov_0.943128 | non-coding | 99.654 | 289 | 1 | 288 | 17425 | 17137 | 1.64E-152 |
| Amar10 | 1 | NODE_68702_length_1499_cov_17.618143 | ND6-ND4l-G-ND2(p) | 99.733 | 1499 | 1 | 1499 | 1496 | 1 | 0 |
|  | 2 | NODE_128195_length_985_cov_3.558370 | ND2(p)-D-T-12s(p) | 100 | 985 | 1 | 985 | 2591 | 1607 | 0 |
|  | 3 | NODE_6_length_982_cov_2.944751 | 12s(p)-M-C-Y(p) | 100 | 960 | 23 | 982 | 2574 | 3533 | 0 |
|  | 4 | NODE_2_length_3610_cov_3.371356 | Y(p)-S-cox3-F-A-cox1-P-cox2(p) | 99.64 | 3610 | 1 | 3610 | 3530 | 7134 | 0 |
|  | 5 | NODE_344924_length_493_cov_3.127404 | cox2(p) | 99.797 | 493 | 1 | 493 | 7832 | 7340 | 0 |
|  | 6 | NODE_1_length_4824_cov_3.021066 | cytb(p)-16s-atp8-ND4-H-E-S-atp6-ND3(p) | 99.979 | 4824 | 1 | 4824 | 7947 | 12770 | 0 |
|  | 7 | NODE_17581_length_3787_cov_2.660199 | ND3(p)-Q-I-K-L-ND1-V-N-ND5-L-W | 99.947 | 3787 | 1 | 3787 | 12830 | 16615 | 0 |
|  | 8 | NODE_555236_length_353_cov_1.880435 | non-coding | 100 | 353 | 1 | 353 | 16808 | 17160 | 0 |
|  | 9 | NODE_11_length_196_cov_6.252101 | non-coding | 98.98 | 196 | 1 | 196 | 17543 | 17738 | 0 |
| A5 | 1 | NODE_6945_length_931_cov_3.593677 | 16s-Cytb | 99.893 | 931 | 1 | 931 | 9075 | 8145 | 0 |
|  | 2 | NODE_35786_length_442_cov_1.479452 | cox1 | 99.548 | 442 | 1 | 442 | 5215 | 5656 | 0 |
|  | 3 | NODE_52165_length_367_cov_1.089655 | ND6 | 99.286 | 140 | 228 | 367 | 638 | 777 | 3.95E-70 |
| A6 | 1 | NODE_450395_length_272_cov_0.748718 | cytb | 100 | 272 | 1 | 272 | 8367 | 8638 | 2.60E-145 |
|  | 2 | NODE_693377_length_195_cov_2.466102 | non-coding | 99.487 | 195 | 1 | 195 | 63 | 257 | 5.40E-101 |
| Aoku2 | 1 | NODE_1578_length_17549_cov_8.766140 | complete 37 genes | 92.393 | 17038 | 31 | 17036 | 279 | 17276 | 0 |

**Table S10 Number of UCEs captured from sequencing data.** v1, UCEs match to v1 probe set; v2, UCEs match to v2 probe set.

| ID | Species | No. UCEs v1 | No. UCEs v2 | Total |
| --- | --- | --- | --- | --- |
| Amar1 | *Archivesica marissinica* | 36 | 16 | 52 |
| Amar2 | *Archivesica marissinica* | 156 | 74 | 230 |
| Amar3 | *Archivesica marissinica* | 15 | 15 | 30 |
| Amar4 | *Archivesica marissinica* | 70 | 53 | 123 |
| **A2** | ***Archivesica marissinica*** | **85** | **57** | **142** |
| **A3** | ***Archivesica marissinica*** | **650** | **351** | **1001** |
| **A4** | ***Archivesica marissinica*** | **410** | **234** | **644** |
| Amar9 | *Archivesica marissinica* | 0 | 0 | 0 |
| **A5** | ***Archivesica marissinica*** | **583** | **343** | **926** |
| Amar6 | *Archivesica marissinica* | 4 | 8 | 12 |
| **A6** | ***Archivesica marissinica*** | **82** | **63** | **145** |
| **A7** | ***Archivesica marissinica*** | **85** | **67** | **152** |
| Amar8 | *Archivesica marissinica* | 9 | 11 | 20 |
| Amar10 | *Archivesica marissinica* | 23 | 28 | 51 |
| Amar11 | *Archivesica marissinica* | 2 | 1 | 3 |
| Anan1 | *Archivesica nanshaensis* | 12 | 10 | 22 |
| Anan2 | *Archivesica nanshaensis* | 1 | 3 | 4 |
| Anan3 | *Archivesica nanshaensis* | 2 | 3 | 5 |
| Aoku2 | *Archivesica okutanii* | 74 | 39 | 113 |
| Aoku1 | *Archivesica okutanii* | 94 | 51 | 145 |
| A_diagonalis | *Archivesica diagonalis* | 24 | 18 | 42 |
| A_gigas | *Archivesica gigas* | 34 | 26 | 60 |
| Pliocardia_sp | *Pliocardia* sp. Blake Spur | 49 | 28 | 77 |
| P_soyoae | *Archivesica soyoae* | 21 | 18 | 39 |
| C_magnifica | *Turneroconcha magnifica* | 72 | 83 | 155 |
| C_fausta | *Calyptogena fausta* | 864 | 497 | 1361 |
| C_extenta | *Archivesica extenta* | 176 | 88 | 264 |
| C_pacifica | *Calyptogena pacifica* | 51 | 19 | 70 |
| Crec | *Calyptogena rectimargo* | 34 | 19 | 53 |
| A_phaseoliformis | *Abyssogena phaseoliformis* | 16 | 12 | 28 |
| A_southwardae | *Abyssogena southwardae* | 21 | 12 | 33 |
| ecnat | *‘Calyptogena’ nautilei* | 354 | 318 | 672 |
| artst | *Archivesica tsubasa* | 64 | 84 | 148 |
| arkut | *Archivesica magnocultellus* | 155 | 233 | 388 |
| phnat | *Archivesica nankaiensis* | 101 | 164 | 265 |
| eclat | *Archivesica laubieri* | 51 | 60 | 111 |
| arkat | *Archivesica kawamurai* | 156 | 253 | 409 |
| plcrt | *Pliocardia crenulomarginata* | 56 | 74 | 130 |
| plsat | *‘Waisiuconcha’* aff. *katsuae* | 54 | 90 | 144 |
| iskat | *‘Vesicomya’ kaikoae* | 29 | 34 | 63 |
| plhat | Pliocardiinae gen. et sp. indet. Haima | 336 | 329 | 665 |

**Figure S1 Photos of fresh clam shells.** a-d, Amar2, length=9.7 cm; e-h, Amar3, length=12.6 cm; i-m, Amar4, length=14.1 cm. Left and right sides, two sides of the same valve.

**Figure S2 Photos of ancient shells (*Archivesica marissinica*).** a-b, Amar5, length=11.3 cm; c-d, Amar6, length=9 cm; e-h, Amar7, length=5.5 cm. Left and right sides, two sides of the same valve.

**Figure S3 Photos of ancient shells (*Archivesica marissinica*).** a-d, Amar10, length=10.7 cm; e-h, Amar11, length=8.8 cm. Left and right sides, two sides of the same valve.

**Figure S4 Photos of ancient shells (*Archivesica okutanii*).** a-d, Aoku2, length=11.7 cm; e-h, Aoku1, length=12.6 cm. Left and right sides, two sides of the same valve.

**Fig. S5 Normalized reads similarity percentage between *Archivesica marissinica* samples based on reads mapped to the nuclear genome.** WGS, Whole genome sequencing. Ama, tissue data from the reduced genome survey data of *A. marissinica* (randomly selected 10 Gb data from SRR11675938).

**Fig S6 Sequencing depth of fresh shell reads in target UCE loci (*A. marissinica*).**

 **Fig S7 Sequencing depth of ancient shell reads in target UCE loci (*A. marissinica*).**

**Fig. S8 DNA damage analysis results for *A. marssinica* fresh shells using bbmap-reads.** No PMD score filtration performed.

**Fig. S9 DNA damage analysis results for *A. marssinica* ancient shells using bbmap-reads.** PMD=1 filtration performed.

**Fig. S10 DNA damage analysis results for *A. marssinica* fresh shells using bwa-mem2-reads.** No PMD score filtration performed.

**Fig. S11 DNA damage analysis results for *A. marssinica* ancient shells using bwa-mem2-reads.** No PMD score filtration performed.

**Fig. S12 DNA damage analysis results for *A. okutanii* shells bbmap-reads (a–d) and bwa-mem2-reads (e–i) using *A. okutanii* transcript as a reference.** a-b, PMD=1 filtration performed; c-d, PMD=3 filtration performed; e-f, no filtration performed; g-h, PMD=3 filtration performed.

**Fig. S13 BBmap-reads length (log2) distribution of fresh and ancient *A. marissnica* shells and *A. okutanii* shells (Aoku1 and Aoku2).**

**Fig. S14 Bwa-mem2-reads length (log2) distribution of fresh and ancient *A. marissnica* shells and *A. okutanii* shells (Aoku1 and Aoku2).**

**Fig. S15 Length fraction of bbmap-mapped reads for DNA damage analysis.** Aoku, *A. okutanii*.

**Fig. S16 Length fraction of bwa-mem2-mapped reads for DNA damage analysis.** Aoku, *A. okutanii*.

**Fig. S17 Length fraction of bbmap-mapped reads for DNA damage analysis without 150-bp reads.** Aoku, *A. okutanii*.

**Fig. S18 Length fraction of bwa-mem2-mapped reads for DNA damage analysis without 150-bp reads.** Aoku, *A. okutanii*.

**Fig. S19 Topology using UCE matrix *Matrix-B* with 1,036 alignments from 25 sequencing data.** Clade with shell DNA data, pink block. Node support value, only presents key nodes.

**Figure S20 Topology using UCE matrix *Matrix-C* with 488 alignments from 43 sequencing data (UCE target capture sequencing, metagenomic sequencing, transcriptome sequencing).** Clade with shell DNA data, pink block. Node support value, only present value < 70.
